# Viral Systemic Movement Is Enhanced by Alteration of a Structural Phloem Protein by the Insect Vector

**DOI:** 10.1002/advs.202506990

**Published:** 2025-09-12

**Authors:** Huijuan Guo, Qingyun Shi, Xiaoyu Ling, Yuting Wu, Lin He, Danyang Li, Keyan Zhu‐Salzman, Yucheng Sun

**Affiliations:** ^1^ State Key Laboratory of Animal Biodiversity Conservation and Integrated Pest Management Institute of Zoology Chinese Academy of Sciences Beijing 100101 China; ^2^ School of Plant Protection Anhui Agricultural University Hefei 230036 China; ^3^ Department of Entomology Texas A&M University College Station TX 77843 USA

**Keywords:** aphid, glucose dehydrogenase, phloem, sieve element occlusion, virus transmission

## Abstract

The ability to access and exploit the vascular system is essential for viral systemic spread within plant hosts. Insect‐borne viruses can be released from the vector's stylets into plant phloem together with saliva components that facilitate viral systemic spread. This study found that *Myzus persicae* released abundant salivary protein glucose dehydrogenase (GLD) into the phloem, establishing an oxidizing environment during feeding, which oxidized cysteine residues in the C‐terminal motif of sieve element occlusion (SEO) proteins. This modification promotes SEO aggregation and directly facilitates interactions between aggregated SEOs and the coat protein of cucumber mosaic virus (CMV), thereby facilitating virus systemic movement in the phloem. When the four cysteine residues of SEO are replaced by serines, aphid GLD can no longer initiate SEO aggregation or accelerate virus systemic infection. These findings reveal a novel function of aphid GLD in accelerating systemic movement of CMV in the plant via oxidizing plant SEO proteins. This biochemical cascade allows viruses to exploit the host vascular system for systemic movement. This work identifies a crucial target in the phloem for mitigating virus infection efficiency after aphid inoculation.

## Graphical abstract

Aphids release salivary protein GLD into plants, which induces ROS in the phloem. This leads to the intermolecular disulfide bonds formation among SEO proteins, promoting their transition from the dispersed state to the aggregated state. SEO can interact with CMV CP. The aggregated SEO proteins then load the viruses to pass through the sieve plate, thereby facilitating their long‐distance movement.

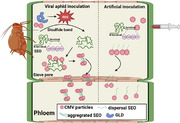

## 1 Introduction

Plants and animals employ fundamentally different strategies to transport fluids, chemicals, and macromolecules, yet they share remarkable structural similarities in their vascular conduits, such as blood vessels and phloem.^[^ ^1^ ^]^ Occlusion is an efficient mechanism for vascular networks to respond to systemic injuries.^[^ ^2^, ^3^ ^]^ In plants, the phloem tissue contains specialized fibril‐like structural proteins that form plugs at sieve plates to block further translocation of photo‐assimilates after injury.^[^ ^3^, ^4^ ^]^ Phloem occlusion serves as a fast‐reacting physical barrier to eliminate the spread of phloem‐infecting pathogens.^[^ ^5^ ^]^ To counteract host plant immunity, viral proteins could either mimic phloem proteins by forming aggregated vesicles or directly utilize phloem proteins as a viral RNA carrier to pass through sieve plates for systemic infection.^[^ ^6^, ^7^ ^]^ Nevertheless, it remains unclear if phloem proteins that function in regulating phloem occlusion are beneficial or detrimental to the systemic infection of plant virus.

Virus dissemination within plants engages two primary routes: cell‐to‐cell movement through plasmodesmata, and long‐distance systemic trafficking through the vascular system.^[^ ^8^, ^9^ ^]^ The short‐distance cell‐to‐cell movement requires modification of plasmodesmata size by viral factors such as movement proteins, but this process is rather slow (2 h per cell). In contrast, long‐distance movement in phloem can reach a few centimeters per hour, favoring virus infection.^[^ ^10^, ^11^ ^]^ In nature, many plant viruses are primarily transmitted by insect vectors. The probing and salivation processes are necessary for the inoculation of plant virus. In this context, some salivary proteins from insect vectors may offer great convenience for virus systemic transport by modifying plant defense or phloem physiology.^[^ ^12^ ^]^ Previous study has found that a carbonic anhydrase excreted by aphids is able to accelerate cell‐to‐cell movement of virus during the post‐transmission infection stage,^[^ ^13^ ^]^ but it is still unclear whether certain salivary proteins could promote virus systemic movement by preventing phloem occlusion.

The plant vasculature is composed of non‐living xylem for water and mineral conduction, and living phloem for sugar and macromolecule transport.^[^ ^14^ ^]^ Sieve tubes, the conduits of the phloem, consist of elongated cells, named sieve elements (SEs), which are connected by perforated sieve plates. Pores on the sieve plate facilitate the sap flow between the SEs.^[^ ^14^, ^15^ ^]^ Free flow of phloem sap inside the sieve tubes not only provides a continuous source of photo‐assimilates for phloem‐feeding insects but also furnishes a “highway” for virus systemic infection.^[^ ^12^ ^]^ SEs of angiosperms contain abundant structural phloem proteins (P‐proteins) that are characterized by their tubular, fibrillar or granular ultrastructure. P‐proteins are mainly encoded by the *sieve element occlusion* (*SEO*) gene family, and are categorized into non‐dispersive and dispersive types.^[^ ^4^, ^16^ ^]^ Non‐dispersive type P‐proteins, also named forisomes, are found exclusively in leguminous plants that undergo a remarkable anisotropic conformational change from unique spindle‐shaped P‐proteins to a three‐ to nine‐fold bigger plug‐like complex, in response to various stimuli such as wounding and Ca^2+^ flux.^[^ ^17^, ^18^ ^]^ Upon infestation by aphids, the spindle‐shaped state of forisomes, instead of plug‐like complexes, remains in the phloem due to Ca^2+^‐binding proteins in aphid watery saliva.^[^ ^19^ ^]^ In contrast, dispersive type P‐proteins, existing in all dicot plants, lack the spindle‐shaped state. Transmission electron microscopy evidence shows that dispersive P‐proteins exhibit two states: agglomeration with fibril bundles anchored on the SE membrane or a randomly dispersed state with disordered fibrils filling the SE lumen.^[^ ^4^, ^20^, ^21^ ^]^

Dispersive type P‐proteins (hereafter referred to as dispersive SEO proteins) initially form aggregated fibrillar structures in immature SEs, which transition to a dispersed state as SEs mature.^[^ ^22^ ^]^ In mechanically injured plants, the dispersive SEO proteins plug sieve plates to prevent the loss of photo‐assimilates. This sealing function is supported by a case study in *Nicotiana tabacum*, where knockdown of two *SEO* genes significantly increased phloem exudation. Considering that both aggregated and dispersed forms are simultaneously observed at occlusion sites, it remains unclear which form of SEO proteins is responsible for the phloem occlusion.^[^ ^20^ ^]^ In contrast, in uninjured plants, both forms can span sieve plates without blockage of sieve pores.^[^ ^4^ ^]^ Aphid feeding usually causes minimal physical damage to SEs relative to chewing insects or mechanistic wounding. It is speculated that dispersive SEO proteins remain non‐occlusive, allowing phloem transport to proceed without physical blockage when infested by aphids. This is in agreement with the observations that aphids exhibit reduced feeding efficiency on *Arabidopsis SEO* knockdown mutants.

SEO proteins contain a conserved C‐terminal motif M1 that is characterized by four cysteine residues.^[^ ^23^ ^]^ These cysteine residues of SEO proteins are likely to form intermolecular disulfide bridges by oxidation.^[^ ^23^, ^24^, ^25^ ^]^ It is also conceived that the transition from dispersed state to aggregated state for dispersive SEO proteins is initiated by the formation of intermolecular disulfide bonds under an oxidative environment. Moreover, dispersive SEO proteins in *Arabidopsis* and *N. tabacum* contain an intrinsically disordered region (IDR) at the N‐terminus.^[^ ^23^ ^]^ Weak and multivalent protein‐protein interactions can be formed between the IDRs of two different molecules, in which proteins usually undergo liquid‐liquid phase separation (LLPS), and transition from a dispersed state to an aggregated state through IDR‐mediated assembly.^[^ ^26^ ^]^ In plants, LLPS can promote the co‐aggregation of certain proteins with virus particles by binding to viral DNA/RNA or viral proteins.^[^ ^27^ ^]^ In the light of this feature, we presume that SEO proteins are likely to facilitate long‐distance movement of plant viruses in phloem by co‐aggregating with viruses.

As a common arthropod vector, *Myzus persicae* is capable of transmitting more than 100 plant viruses, including cucumber mosaic virus (CMV), most of which are transmitted in a non‐persistent manner. CMV particles are transiently attached to the tip of aphid stylets and delivered into plant tissues during aphid probing and salivation.^[^ ^28^ ^]^ Widespread evidence shows that aphid saliva triggers reactive oxygen species (ROS) accumulation in phloem, thereby establishing an oxidative environment at the feeding site.^[^ ^29^ ^]^ We therefore hypothesize that the oxidative environment in the phloem established by aphid salivary proteins triggers the formation of intermolecular disulfide bonds via oxidizing the cysteine residues at the C‐terminus of SEO proteins. This promotes the transition of SEO from the dispersed state to the aggregated state, thereby co‐aggregating with CMV to facilitate virus translocation across the sieve pores. To experimentally test these hypotheses, we specifically investigated the following: (i) identification of the aphid salivary protein(s) that trigger an oxidative environment in phloem; (ii) whether the formation of intermolecular disulfide bonds among SEO proteins promotes transition from the dispersed state to the aggregated state under an oxidative environment; and (iii) whether the aggregation of SEO facilitates interaction with CMV to efficiently promote systemic movement of the virus.

## 2 Result

### 2.1 Aphid Salivary Protein GLD Facilitated Virus Systemic Infection

**Figure 1:**
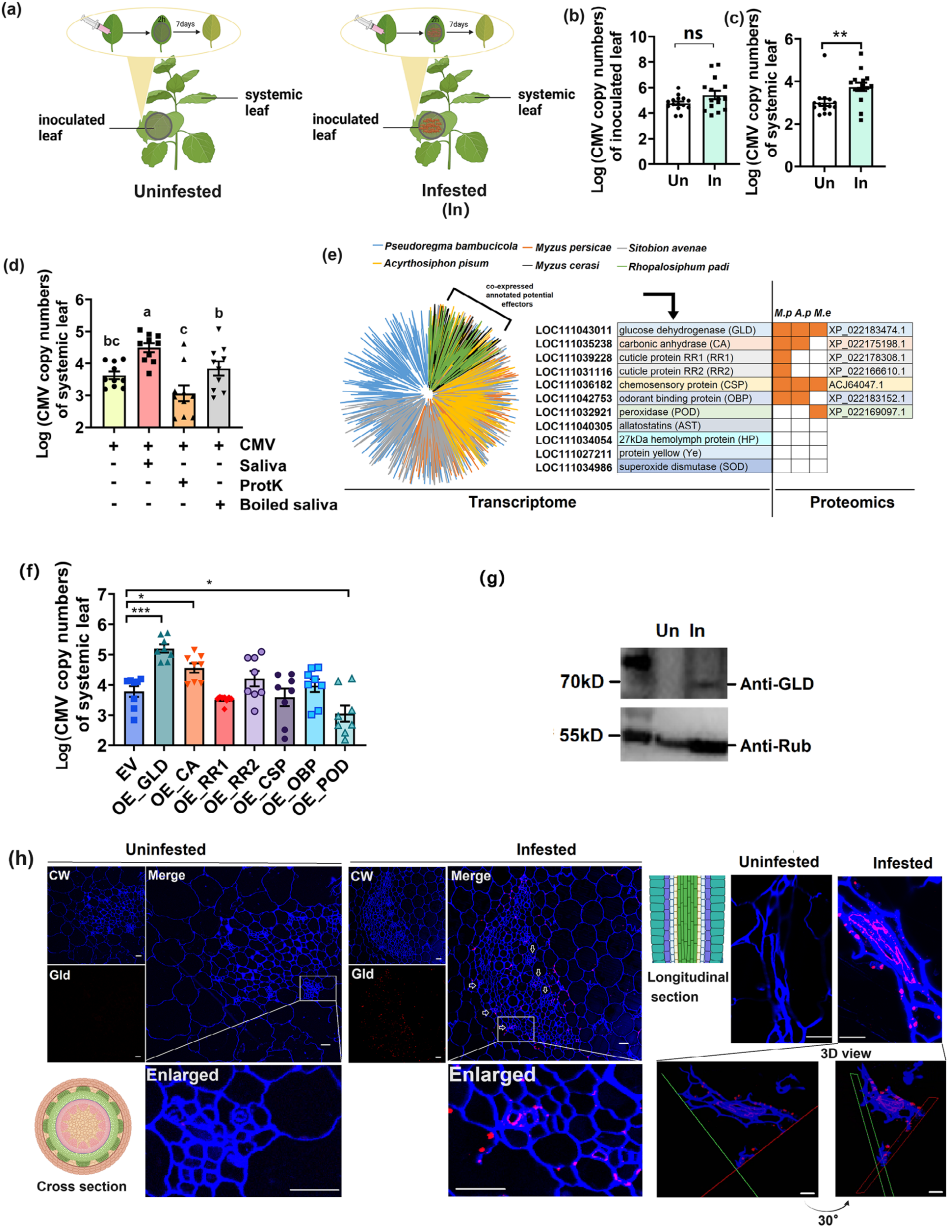
Secreted salivary protein glucose dehydrogenase (GLD) of aphid facilitated systemic movement of virus. a) Schematic representation of the artificial inoculation of the virus on plant leaves with and without aphid feeding. b,c) The CMV copy numbers in local and systemic leaves in CMV‐inoculated plants with and without aphid feeding (*n* = 15, Student's *t* test, ^**^ *p *< 0.01). Data are presented as means ± SE. ns, no significant difference. d) The CMV copy numbers in systemic leaves were quantified 7 days after co‐infiltrating the same amount of CMV particles with either untreated aphid saliva, proteinase K‐treated saliva, or boiled saliva (*n* = 15, one‐way ANOVA). Data are presented as means ± SE. Means with different letters represented significant differences among treatments, as determined by Tukey's post‐hoc test at *p* < 0.05. e) Profiling of salivary transcriptomes from six aphid species and saliva proteomics from three aphid species identified 7 conserved candidate saliva effectors in aphids that could potentially be secreted into plants. *M.p*., *Myzus persicae*; *A.p*., *Acyrthosiphon pisum*; *M.e*., *Macrosiphum euphorbiae*. f) CMV copy numbers of systemic leaves of plants that overexpressed 7 candidate aphid saliva effectors respectively 7 days after phloem injection with CMV particles (*n* = 8, Student's *t* test, ^*^ *p *< 0.05, ^***^ *p *< 0.001). Data are presented as means ± SE. (g) Detection of GLD in aphid‐infested leaves by western blot. Lane 1: uninfested leaf; lane 2: aphid‐infested leaf. h) GLD localization images with both cross‐ and longitudinal sections of the vascular system of aphid‐infested leaves. Scale bar, 20 µm. Calcufluor White was used to stain the cell wall. GLD antibody was used to detect aphid‐secreted GLD in plant cells.

Following artificial inoculation of leaves with the virus using a needleless syringe, aphids were allowed to feed on the inoculated leaves to assess the impact of aphid feeding on the spread of CMV. Systemic leaves of aphid‐infested plants had higher CMV copy numbers than uninfested plants (**Figure** **1**a–c). We therefore presumed that aphid salivary effectors promote systemic spread of the virus. Co‐infiltration of aphid saliva and CMV increased CMV copy numbers in systemic leaves relative to CMV inoculation. Reduced CMV copy numbers via proteinase K treatment or boiled saliva suggested that some salivary proteins could promote CMV infection (Figure 1d).

To identify the putative salivary effector protein(s) that are conserved among aphid species, we analyzed published databases of salivary gland transcriptome from six aphid species including *Pseudoregama bambucicola*, *M. persicae*, *Myzus cerasi*, *Sitobion avenae*, *Rhopalosiphum padi*, and *Acyrthosiphon pisum*.^[^ ^29^, ^30^, ^31^, ^32^, ^33^, ^34^ ^]^ A total of 636, 455, 459, 104, 106, and 329 salivary gland‐expressed genes were annotated in each aphid species, respectively. Using a presence/absence filtering approach, salivary protein genes that were conserved across all six aphid species were screened. The following excel formula was used to determine which genes were present in all six datasets: = IF (AND (COUNTIF (B:B,A1) >0, COUNTIF (C:C,A1) >0, COUNTIF (D:D,A1) >0, COUNTIF (E:E,A1) >0, COUNTIF (F:F,A1) >0), “Yes”, “No”). Genes marked with “Yes” were identified in all six species. These candidates were further filtered by the presence of signal peptides and the lack of transmembrane domains, which were the common selective criteria for salivary proteins. Eventually, 11 homologous salivary protein genes shared among the six aphid species were identified. Furthermore, saliva proteomic databases of three aphid species, including *M. persicae*, *A. pisum*, and *Macrosiphum euphorbiae* were used to validate whether these 11 candidates existed in aphid saliva.^[^ ^29^, ^33^, ^35^ ^]^ Seven candidate genes in *M. persicae* were screened out by at least one hit on three saliva proteomes. They were listed below: LOC111043011 (glucose dehydrogenase, GLD), LOC111035238 (carbonic anhydrase, CA), LOC111039228 (cuticular protein RR1), LOC111031116 (cuticular protein RR2), LOC111036182 (chemosensory protein, CSP), LOC111042753 (odorant binding protein, OBP), and LOC111032921 (peroxidase, POD). It was indicated that these seven proteins were potentially released into plants during aphid feeding (Figure 1e). qPCR results confirmed that the gene transcripts of the seven selected proteins were expressed in the salivary glands of *M. persicae*, of which GLD had the highest transcript abundance (Figure S1a, Supporting Information). These seven genes of *M. persicae* were expressed in *N. tabacum* to evaluate their effects on virus systemic infection (Figure S1b, Supporting Information). Transient expression of *GLD* and *CA* in plants increased CMV copy numbers by 26‐ (*p *< 0.001) and four‐fold (*p *< 0.05), respectively, when compared with the empty vector (EV) plants. Expression of POD decreased CMV copy numbers by five‐fold (*P*<0.05) in systemic leaves (Figure 1f). Since GLD was identified in all three aphid species, and resulting in the strongest virus systemic infection, we decided to further investigate the molecular function of aphid GLD in virus systemic infection. Western blotting analysis only detected GLD in aphid‐infested plant leaves (Figure 1g; Figure S2a, Supporting Information). Moreover, both cross‐ and longitudinal sections of the vascular system of aphid‐infested leaves demonstrated that aphid GLD could be secreted into the phloem of plants (Figure 1h; Figure S1c and Movie S1, Supporting Information).

### 2.2 GLD Increassed ROS Production in the Phloem

**Figure 2:**
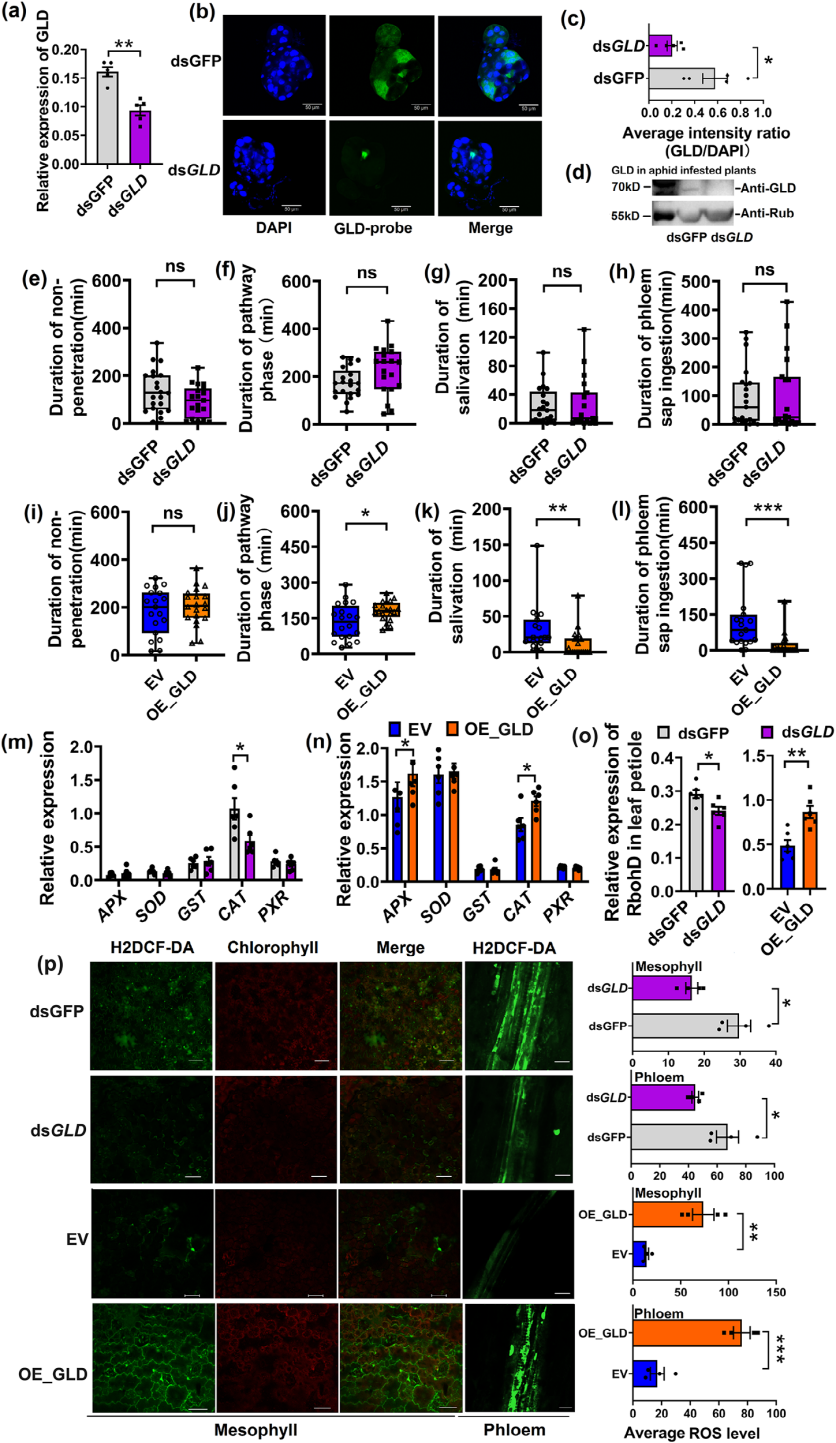
Aphid‐derived GLD induced ROS in phloem. a) Knockdown of GLD in *M. persicae* reduced GLD transcripts in the salivary gland (*n* = 5, Student's *t* test, ^**^ *p *< 0.01). Data are presented as means ± SE. (b&c) RNA FISH localization showing a lower expression of *GLD* in the dissected salivary gland of ds*GLD* aphids relative to those ds*GFP* aphids (*n* = 4, Student's *t* test, ^*^ *p *< 0.05). Data are presented as means ± SE. Scale bar, 50 µm. d) Detection of GLD in ds*GLD* or ds*GFP* aphid‐infested leaves by western blot. Lane 1: ds*GFP* aphid‐infested leaf; lane 2: ds*GLD* aphid‐infested leaf. e–h) Feeding activities, including time spent in non‐penetration, pooled pathway phase activities in the intercellular space of epidermis and mesophyll cells, salivation time, phloem sap ingestion of ds*GLD* or ds*GFP* aphids during an 8‐h feeding period using electronic penetration graph (*n* = 20, Mann‐Whitney U test). Data are presented as means ± SE. ns, no significant difference. i–l) Feeding activities including time spent in non‐penetration, pooled pathway phase activities in intercellular space of epidermis and mesophyll cells, salivation time, phloem sap ingestion of aphids when fed on EV‐infiltrated or GLD‐overexpressed plants during an 8 h feeding period (*n* = 20, Mann‐Whitney U test, ^*^ *p *< 0.05, ^**^ *p *< 0.01, ^***^ *p *< 0.001). Data are presented as means ± SE. ns, no significant difference. m) Relative expression levels of ROS‐related genes in plant leaves when infested by ds*GFP* and ds*GLD* aphids (*n* = 6, Student's *t* test, ^*^ *p *< 0.05), and n) when associated with EV‐infiltration and GLD‐overexpression (*n* = 6, Student's *t* test, ^*^ *p *< 0.05). Data are presented as means ± SE. o) Relative gene expression of ROS synthetic enzyme *respiratory burst oxidase homolog protein D* (*RbohD*) in leaf petiole (including phloem) of ds*GFP* versus ds*GLD* aphid‐infested leaves, and those of *GLD* overexpressed versus empty vector inoculated plants (*n* = 4, Student's *t* test, ^*^ *p *< 0.05, ^**^ *p* < 0.01). Data are presented as means ± SE. p) Localization of ROS fluorescent probe in mesophyll and phloem of ds*GFP* versus ds*GLD* aphid‐infested leaves, and *GLD* overexpressing versus empty vector‐inoculated leaves (*n* = 4, Student's *t* test, ^*^ *p *< 0.05, ^**^ *p *< 0.01, ^***^ *p *< 0.001). Data are presented as means ± SE. Scale bar, 20 µm.

GLD has been identified as one of the most commonly detectable salivary proteins across various insect species and acts as a suppressor for plant defenses.^[^ ^36^, ^37^ ^]^ It was hypothesized that the absence of GLD could impair the phloem feeding efficiency of aphids. Forty‐eight hours after ds*GLD* injection, ds*GLD* injection reduced GLD transcripts in the salivary gland by 43% relative to ds*GFP*‐injected aphids (**Figure** **2**a). This result was further confirmed by RNA fluorescent in situ hybridization (FISH) localization on dissected salivary glands (Figure 2b,c), as well as by western blot detection of GLD in leaves when infested with ds*GLD*‐ and ds*GFP*‐injected aphids (Figure 2d; Figure S2b, Supporting Information). To determine the role of GLD in the feeding activity of aphids, an electrical penetration graph (EPG) was used to compare the feeding behavior of ds*GLD*‐ and ds*GFP*‐injected aphids. Little difference was observed in time spent in non‐penetration, pathway phase, salivation, and phloem feeding between ds*GLD*‐ and ds*GFP*‐injected aphids (Figure 2e–h). However, aphids fed on GLD‐infiltrated *N. tabacum* plants spent more time in the pathway phase but spent less time on salivation and phloem feeding than those fed on EV‐infiltrated plants (Figure 2i–l). The reduced feeding efficiency was possibly attributed to the ubiquitous expression of aphid GLD in agro‐infiltrated leaf tissue. Instead, it should primarily be secreted into sieve tubes during aphid feeding. Since GLD is an oxidoreductase, it was still unclear whether aphid GLD could induce ROS production in plants. The expressions of ROS‐related genes were determined by q‐PCR. Infestation of ds*GLD*‐injected aphids reduced the expression of *catalase* (*CAT*) and *RbohD* in plants compared with ds*GFP*‐injected aphids (Figure 2m,o). Plants overexpressing *GLD* significantly up‐regulated *ascorbate peroxidase* gene (*APX*) and *CAT* in leaves as well as *RbohD* in leaf petiole, when compared with EV‐infiltrated plants (Figure 2n,o). Furthermore, both aphid‐infested plants and GLD‐expressed plants that were infiltrated with fluorescent dye precursor (H_2_DCF‐DA) showed GLD‐activated H_2_O_2_ production in both mesophyll and phloem (Figure 2p).

### 2.3 SEOs Were Differentially Expressed in *N. tabacum* When Associated With Aphid Infestation, Saliva Infiltration and GLD‐Overexpression

**Figure 3:**
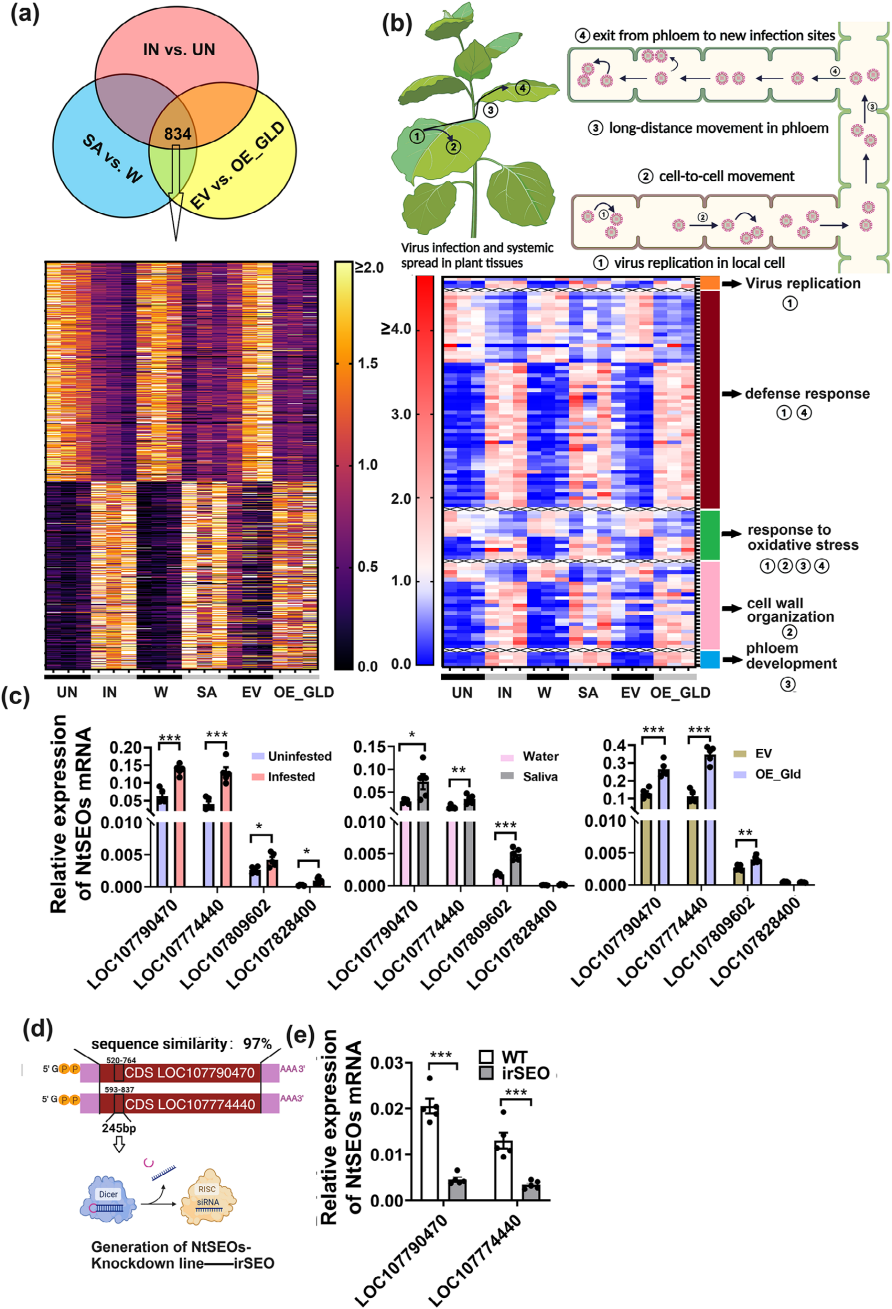
Aphid infestation, saliva infiltration, and overexpression of GLD upregulated sieve element occlusion genes in *N. tabacum*. a) Venn diagram and heat map showing differentially expressed genes (DEGs) in comparisons of uninfested (UN) versus aphid‐infested wild‐type plants (IN), water‐infiltrated (W) versus aphid saliva‐infiltrated wild‐type plants (SA), and EV‐infiltrated (EV) versus GLD‐expressed plants (OE_GLD). Three biological replicates were performed for RNA‐seq. b) A schematic representation illustrating virus systemic spread processes and the involvement of DEGs: ① virus replication in local cells, ② cell‐to‐cell movement, ③ long‐distance movement in phloem, and ④ exit from phloem to new infection sites. The heat map showed 834 overlapping DEGs from Venn diagram, of which 102 were involved in the process of virus replication and systemic spread. Specifically, these DEGs were enriched in the pathways of virus replication, defense response, response to oxidative stress, cell wall organization, and phloem development. Among the phloem development‐associated genes, 4 *sieve element occlusion* (*SEO*) genes were up‐regulated when treated with aphid infestation, saliva infiltrated and *GLD*‐overexpression. c) The gene transcripts of four *SEOs* (*LOC107790470*, *LOC107774440*, *LOC107809602*, and *LOC107828400*) were determined by qPCR when treated with aphid infestation, saliva infiltration and overexpression of GLD (*n* = 5, Student's *t* test, ^*^ *p *< 0.05, ^**^ *p *< 0.01, ^***^ *p *< 0.001). Data are presented as means ± SE. d) The coding sequence (CDS) from *LOC107790470* and *LOC107774440* had high similarity. A hairpin RNA was designed to target a conserved 245‐bp CDS fragment from *LOC107790470* (520‐764 bp) and *LOC107774440* (593‐837 bp), leading to the subsequent generation of *NtSEO‐RNAi* line (*irSEO*). e) *irSEO* plants exhibited lower gene expression of *LOC107790470* and *LOC107774440* than wild‐type plants (*n* = 5, Student's *t* test, ^***^ *p *< 0.001). Data are presented as means ± SE.

RNA‐seq analyses were performed to compare the transcriptome profiles in aphid‐infested versus uninfested wild‐type plants, aphid saliva‐infiltrated versus water‐infiltrated wild‐type plants, and in EV‐infiltrated plants versus GLD‐expressed plants. A total of 7899, 4242, and 4325 differentially expressed genes (DEGs) were identified in the treatments of aphid infestation, saliva infiltration, and GLD‐expression when compared with their corresponding controls, respectively. Among them, 834 DEGs were shared in common across all three comparison groups (**Figure** **3**a). Since the treatments of aphid infestation, saliva infiltration, and GLD‐overexpression could promote CMV systemic infection, 102 out of 834 overlapping DEGs with functions related to virus replication, defense response, response to oxidative stress, cell wall organization, and phloem development were primarily focused (Figure 3b). Remarkably, 4 DEGs associated with phloem development, including *LOC107790470*, *LOC107774440*, *LOC107809602*, and *LOC107828400*, were annotated to encode SEO proteins. As a matter of fact, there are 10 annotated SEOs in N. tabacum, and 4 of them were screened out by the above RNA‐seq analyses, and were upregulated when infested by aphids. qPCR results confirmed that aphid infestation, saliva infiltration, and overexpression of *GLD* upregulated the expression of *LOC107790470*, *LOC107774440*, and *LOC107809602* in plants (Figure 3c). The expression levels of *LOC107790470* and *LOC107774440* were significantly higher than those of *LOC107809602* and *LOC107828400* in *N. tabacum* (Figure 3c). Moreover, wild‐type plants infested with ds*GLD*‐injected aphids exhibited lower expression levels of *LOC107790470* and *LOC107774440* than those infested with ds*GFP*‐injected aphids (Figure S3a, Supporting Information). *LOC107790470* was previously characterized as SEO1 in *N. tabacum* ^[^ ^20^ ^]^ and share 97% similarity of CDS sequence with *LOC107774440*. *NtSEO‐RNAi* lines (*irSEO*) were generated with a silencing target on a conserved 245 bp region shared by *LOC107790470* and *LOC107774440* (Figure 3d), and *irSEO* plants exhibited lower gene expression of *LOC107790470* and *LOC107774440* than wild‐type plants (Figure 3e).

### 2.4 Phloem SEO Proteins Co‐Localized With CMV and Accelerated Virus Systemic Spread

**Figure 4:**
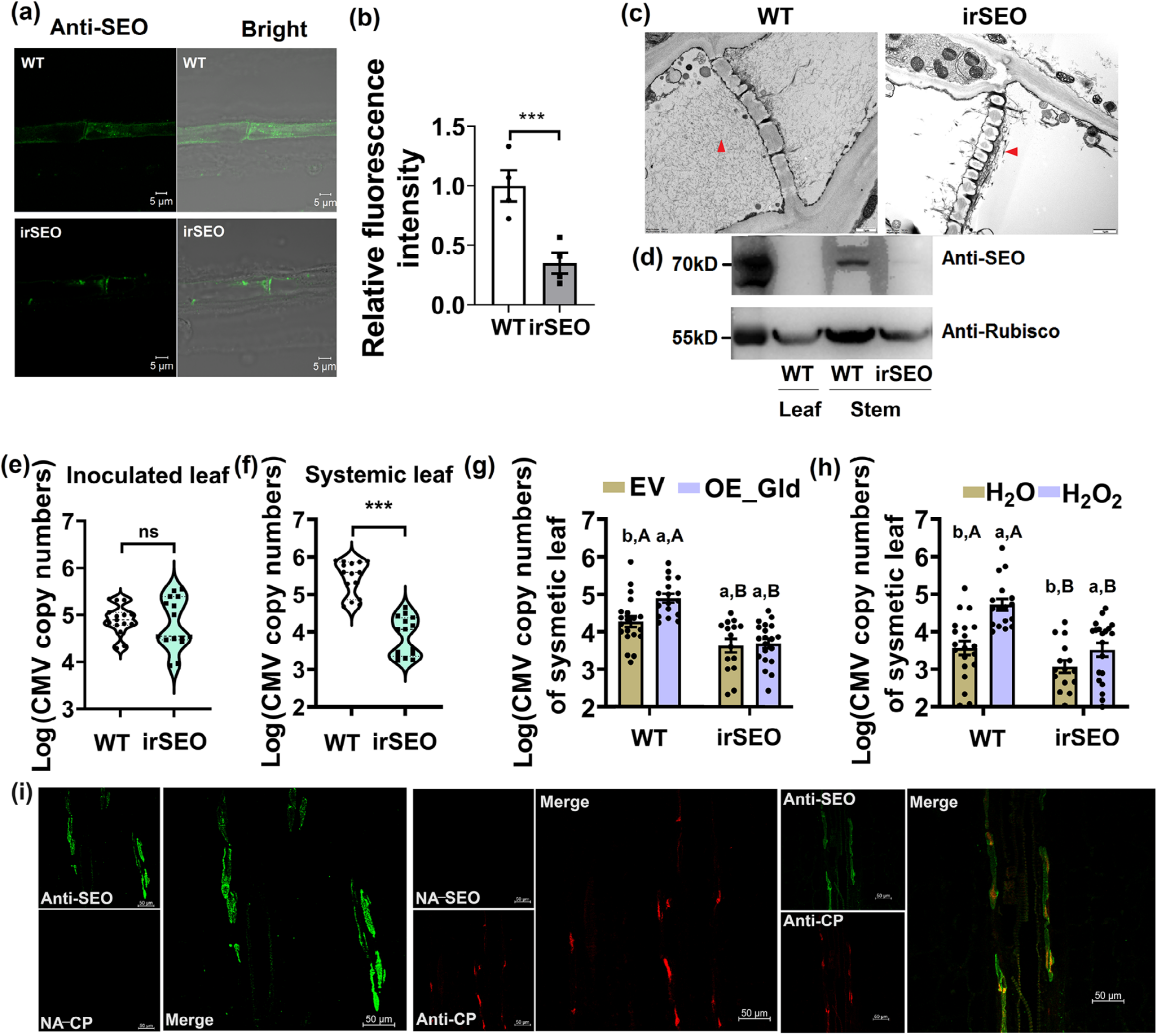
Phloem SEO proteins promoted virus systemic spread within plant. a) Immunofluorescence localization of SEO in wild‐type and *irSEO* plants. The SEO antibody was used to detect SEO in phloem. Scale bar, 5 µm b) The relative fluorescence intensity of SEO in both wild‐type aphid ir*SEO* plants (*n* = 4, Student's *t* test, ^***^ *p *< 0.001). Data are presented as means ± SE. c) Sieve elements of wild‐type and *irSEO* plants were examined by TEM. Filaments in sieve elements were more abundant in wild‐type than *irSEO* plants; Scale bar, 1 µm. The phloem fibrils were indicated by red arrow. d) Detection of SEO in leaves and stems by western blotting analysis, lane 1: wild‐type plant leaf; lane 2: wild‐type plant stem; lane 3: *irSEO* plant stem. e,f) CMV copy numbers in local and systemic leaves of wild‐type and *irSEO* plants (*n* = 15, Student's *t* test, ^***^ *p *< 0.001). Data are presented as means ± SE. ns, no significant difference. g) CMV copy numbers in systemic leaves in response to GLD‐overexpression in wild‐type and *irSEO* plants. Different lowercase letters indicated significant differences between GLD‐overexpression and EV treatment within the same genotype plants, while different uppercase letters indicated significant differences between genotypes, as determined by Tukey's post‐hoc test at *p *< 0.05 (*n* = 20, two‐way ANOVA). Data are presented as means ± SE. h) CMV copy numbers of systemic leaves associated with water and H_2_O_2_‐treated wild‐type and *irSEO* plants. CMV was artificially inoculated. Different lowercase letters indicated significant differences between H_2_O_2_ and H_2_O treatment within the same genotype plants, while different uppercase letters indicated significant differences between two plant genotypes, as determined by Tukey's post‐hoc test at *p *< 0.05 (*n* = 20, two‐way ANOVA). Data are presented as means ± SE. i) Co‐localization of SEO and CMV coat protein (CP) in phloem of CMV‐infected plants. The antibodies of SEO and CMV CP were used to detect SEO and CMV in phloem, respectively. Sections incubated with secondary antibody without application of the primary antibody of SEO or CP served as negative controls (NA). Scale bar, 50 µm.

SEO polyclonal antibody was used to detect SEO proteins, and confocal microscopy showed that SEO protein was distributed in phloem with higher abundance in wild‐type plants relative to *irSEO* plants (**Figure** **4**a,b). Transmission electron microscopy (TEM) images showed that *irSEO* plants were unable to synthesize a normal amount of SEO fibrils in phloem, which possessed much fewer fibrils than wild‐type plants (Figure 4c). Such structural characteristics were consistent with previously reported SEO proteins assembled in tobacco plants.^[^ ^7^ ^]^ Western blotting analysis showed that SEO proteins were expressed in the stem rather than leaves, and the protein level in *irSEO* plants was much lower than in wild‐type plants (Figure 4d; Figure S4, Supporting Information). Aphids fed on *irSEO* plants spent a longer time salivating but a shorter time phloem feeding than those fed on wild‐type plants. The frequency of salivation was increased in aphids associated with *irSEO* plants (Figure S5, Supporting Information). Little difference was observed in CMV copy numbers of inoculated leaves between *irSEO* plants and wild‐type plants when artificially inoculated with the virus. In contrast, *irSEO* plants had lower CMV copy numbers than wild‐type plants in systemic leaves. These results indicated that SEO proteins promoted phloem feeding of aphid and systemic infection of the virus (Figure 4e,f).

Since GLD increased SEO protein expression and promoted CMV systemic infection, the relationship between aphid GLD and SEO protein was further investigated in plants. Overexpression of GLD increased CMV copy number in systemic leaves of wild‐type plants, but not for *irSEO* plants (Figure 4g). Knock‐down of *GLD* in aphids decreased CMV copy numbers in systemic leaves of wild‐type plants, but did not affect CMV copy numbers in *irSEO* plants (Figure S3b, Supporting Information). Likewise, H_2_O_2_‐induced increase in CMV copy numbers was significantly lower in *irSEO* than in wild‐type plants (Figure 4h). These results strongly suggested that SEO protein was essential in the acceleration of CMV systemic infection triggered by aphid GLD. In addition, SEO protein co‐localized with CMV coat protein (CP) in phloem, suggesting a physical interaction between them (Figure 4i).

### 2.5 GLD Induced Aggregation of SEO Proteins in Phloem Near the Sieve Plate

**Figure 5:**
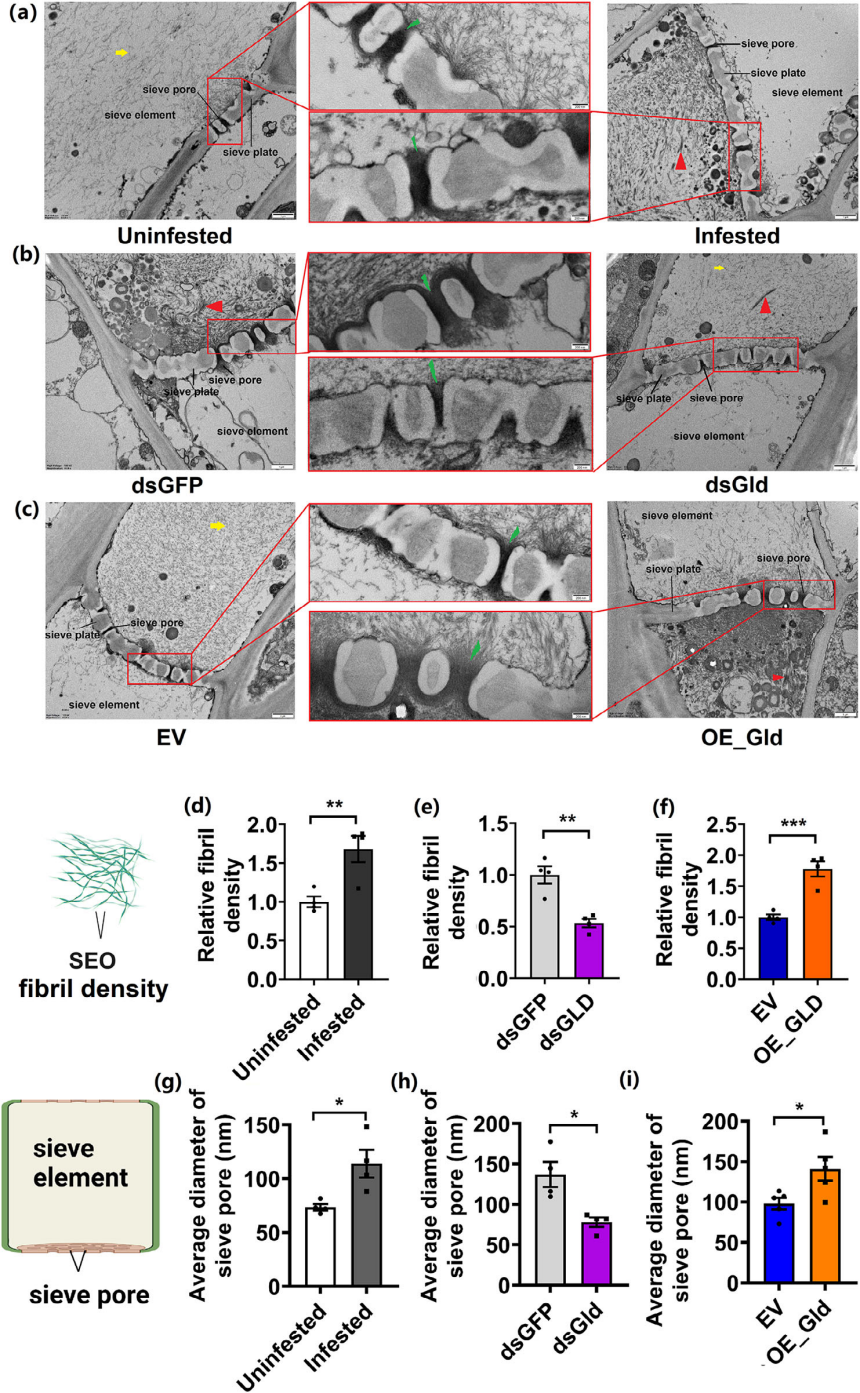
The ultrastructure of phloem proteins determined by TEM. a) Infested by *M. persicae* for 24 h, b) Infested by ds*GFP* and ds*GLD* aphids, c) GLD‐overexpressing plants. The dispersed fibrils were indicated with yellow arrows, and aggregated fibrils were indicated with red arrows. The fibrils traversing sieve pores were marked by green arrows. Scale bar, 1 µm. The region enclosed by the red rectangle was shown at higher magnification. Scale bar, 200 nm. Relative density of fibril in d) aphid‐uninfested and ‐infested leaves, and e) ds*GFP* and ds*GLD* aphid‐infested leaves, and f) EV and GLD overexpressed leaves (*n* = 4, Student's *t* test, ^**^ *p *< 0.01, ^***^ *p *< 0.001). Data are presented as means ± SE. Average diameters of sieve pores in g) uninfested and infested leaves, h) ds*GFP* and ds*GLD* aphid‐infested leaves, and j) EV and GLD‐overexpressing leaves (*n* = 4, Student's *t* test, ^*^ *p *< 0.05). Data are presented as means ± SE.

TEM images of uninfested leaves displayed irregular and randomly oriented dispersed SEO fibril structures distributed in phloem. Upon aphid infestation, dispersed SEO fibril aggregated into highly ordered complexes near sieve plates (**Figure** **5**a,d). Knockdown of *GLD* expression in aphids reduced the aggregation level of SEO proteins (Figure 5b,e). Conversely, GLD‐expressing plants exhibited higher levels of aggregated SEO proteins than control plants (Figure 5c,f). To establish the cause‐effect relationship between GLD‐induced ROS and the aggregation of SEO protein, 1 mm H_2_O_2_ was applied exogenously in the phloem. A higher aggregation level of SEO proteins was observed in H_2_O_2_‐treated plants relative to control plants (Figure S6, Supporting Information). Application of dithiothreitol (DTT) alleviated the aggregation level of SEO proteins caused by H_2_O_2_ (Figure S6, Supporting Information). TEM images also showed that GLD‐induced ROS in phloem could promote the aggregation level of SEO proteins near the sieve plate. SEO localization in phloem exhibited significant methodological dependence. Previous studies demonstrated that excised leaf segments subjected to chemical fixation displayed characteristic SEO protein aggregation at sieve plates, which was due to tissue isolation‐induced metabolic disruption and artificial osmotic shock. In contrast, whole plant fixation was reported to well preserve SEO protein distribution throughout the lumen of the sieve tube.^[^ ^4^ ^]^ The relevant assays were therefore performed using the whole plant fixation method to prevent prefixation artifacts.

Larger average diameters of sieve pores were observed by TEM in the phloem of aphid‐infested plants than in uninfested plants (Figure 5g). As expected, plant infested by *dsGLD‐*injected aphids exhibited a smaller diameter of sieve pores than plants infested by *dsGFP‐*injected aphids. Likewise, GLD‐expressing plants exhibited larger diameters of sieve pores than control plants (Figure 5h,i). Furthermore, TEM images revealed that an abundance of fibrillar SEO proteins were passing through the sieve pores. It was likely that aphid GLD induced the structural transformation of SEO proteins from an irregular and randomly oriented dispersed state to a highly ordered aggregated state, allowing more fibrillar components to cross rather than block the sieve pores.

### 2.6 Cysteine Residues in the C‐Terminal Motif of SEO Proteins Were the Basis for SEO Aggregation Under Oxidative Environment

**Figure 6:**
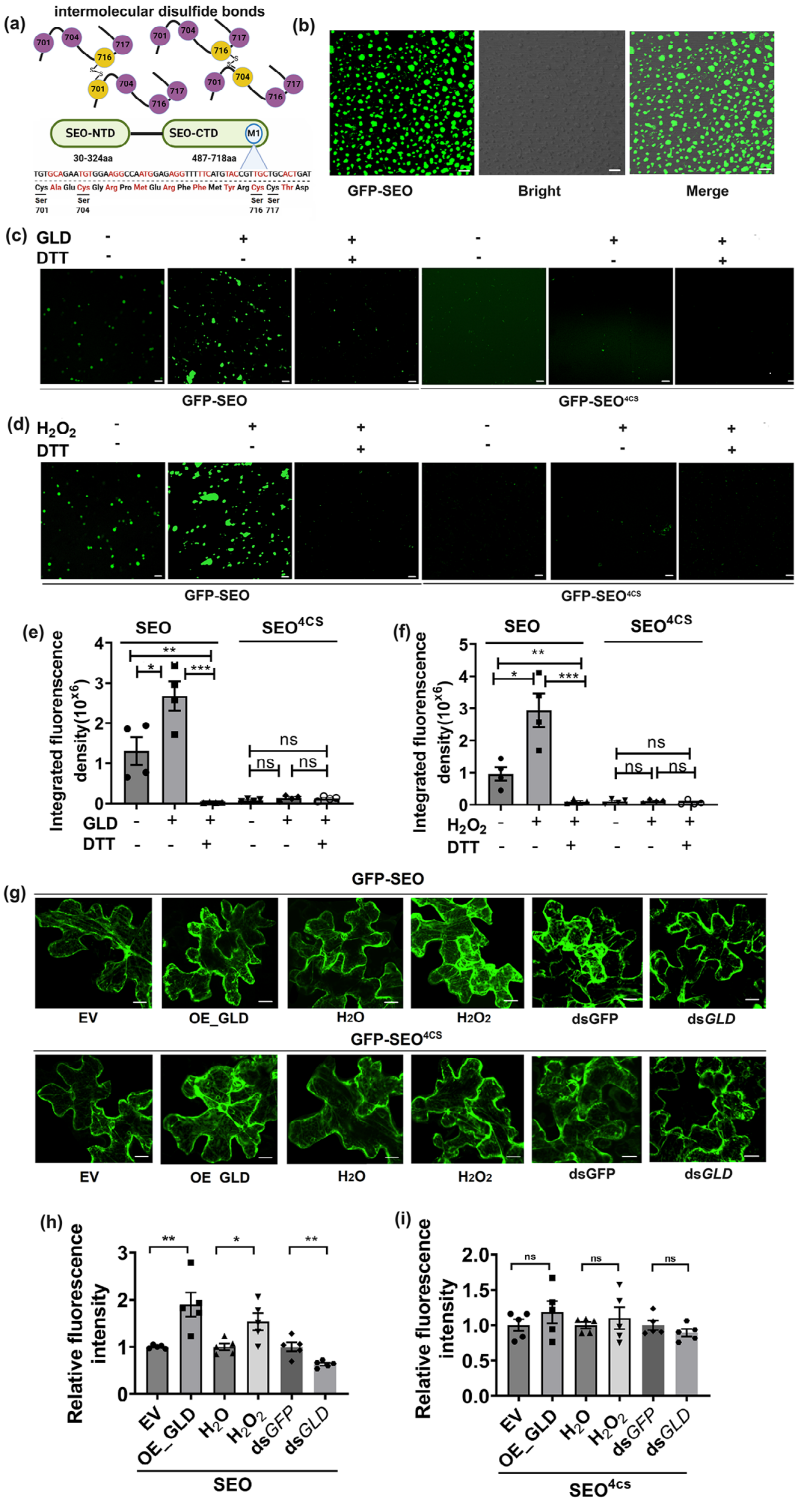
Cysteine residues in the C‐terminal motif of SEO proteins were the basis for SEO aggregation under an oxidative environment. a) Schematic representation illustrating the intermolecular disulfide bonds of the SEO protein. The intermolecular disulfide bonds identified by LC‐MS were formed via cysteine residues C701‐C716 and C704‐C716 crosslinks under native conditions. The SEO protein contained an N‐terminal domain (SEO‐NTD, 30–324aa) and a C‐terminal domain (SEO‐CTD, 487–718aa). The SEO^4CS^ mutant protein was constructed by substituting four cysteines (C701, C704, C716, and C717) at the C‐terminal M1 motif of SEO. b) Purified recombinant GFP‐SEO solution was seen via microscopy formed droplets with various sizes, as observed by microscopy. Scale bar, 10 µm. c–f) Representative confocal microscopy images and quantitative data showing the effects of redox reagents, including GLD, H_2_O_2,_ and DTT, on the formation of aggregates of GFP‐SEO and GFP‐SEO^4CS^ proteins (*n* = 4, Student's *t* test, ^*^ *p *< 0.05, ^**^ *p *< 0.01, ^***^ *p *< 0.001). Data are presented as means ± SE. ns, no significant difference. Scale bar, 10 µm. g–i) SEO aggregation in empty vector‐inoculated leaves versus GLD‐overexpressing leaves, H_2_O versus H_2_O_2_‐infiltrated leaves, ds*GFP*‐ versus ds*GLD* aphid‐infested leaves in *GFP‐SEO* and *GFP‐SEO* ^4CS^ plants (*n* = 5, Student's *t* test, ^*^ *p *< 0.05, ^**^ *p *< 0.01). Data are presented as means ± SE. ns, no significant difference. Scale bar, 10 µm.

SEO proteins consisted of the N‐terminal (SEO‐NTD, 30–324aa) and C‐terminal domains (SEO‐CTD, 487–718aa). Motif M1 in SEO‐CTD has highly conserved 4 cysteine residues (**Figure** **6**a). In the current study, the LC‐MS/MS analysis of purified NtSEO1 protein demonstrated intermolecular disulfide bond formation via C701‐C716 and C704‐C716 crosslinks under native condition, but not under reduced (DTT) condition (Figure 6a; Figure S7, Supporting Information). Since the C‐terminal four cysteine residues (701, 704, 716, and 717) were conserved in most plant species, the SEO^4CS^ mutant protein was constructed, and all four cysteines were substituted with serines.

NtSEO contained a 42aa IDR with low‐complexity domains in the N‐terminus (Figure S8a,b, Supporting Information). Previous work showed that proteins with IDR had the capability to undergo LLPS, which could change from a non‐aggregated phase into a highly condensed aggregate phase.^[^ ^38^ ^]^ It was hypothesized that the phase change of SEO was promoted by the formation of intermolecular disulfide bonds under an oxidative environment, and such aggregation could be further promoted by the weak multivalent interaction among intermolecular IDRs as the protein concentration increased.^[^ ^39^ ^]^ To experimentally test this hypothesis, a standard LLPS‐determination method was used to determine: i) the SEO aggregation when responding to an oxidative environment, and ii) the role of C‐terminal cysteines in the aggregation process. Indeed, increased GFP‐SEO concentration triggered the formation of larger aggregated droplets (Figure S8c, Supporting Information). Individual condensate droplets were photobleached at high laser power, and the fluorescence recovery in the bleached area was measured. Rapid recovery (within 250s) of the fluorescence signals of the bleached droplet (marked by the white arrow) suggested that GFP‐SEO molecules were shifting between condensed and dispersed states (Figure S8d and Movie S2, Supporting Information). In contrast, aggregated droplets were not seen in GFP‐SEO^4CS^ regardless of protein concentration (Figure S8c, Supporting Information). Purified recombinant GFP‐SEO solution formed droplets of various sizes, indicating the coexistence of aggregated and dispersed states of SEO (Figure 6b). Furthermore, either 1 mm H_2_O_2_ or 25 µg mL^−1^ GLD (from Pseudomonas sp., CAS. 9028‐53‐9) induced the aggregates of GFP‐SEO (at 10 µm concentration), while 10 mm reducing reagent DTT diminished such capacity. The oxidation‐induced aggregation was not seen in GFP‐SEO^4CS^ (Figure 6c–f).

SEO aggregation was examined in epidermal cells of the transiently expressed *GFP‐NtSEO* and *GFP‐NtSEO* ^4CS^ *N. tabacum*. SEO aggregation was promoted by GLD overexpression, H_2_O_2_ treatment, and aphid infestation in the epidermal cells of *GFP‐NtSEO* plants, but not in *GFP‐NtSEO* ^4CS^ plants. These results suggested the crucial function of four cysteine residues at C‐terminus in SEO aggregation (Figure 6g–i). It seems that pure SEO proteins in vitro are only able to form droplets, while they tend to form fibril‐like structure in *GFP‐NtSEO* plants. Nevertheless, it cannot be ruled out that the presence of other SEOs or other plant proteins in sieve tubes are potential contributors to this structural difference.

### 2.7 SEO Aggregation Under an Oxidative Environment Promoted Co‐Aggregation With CMV CP

**Figure 7:**
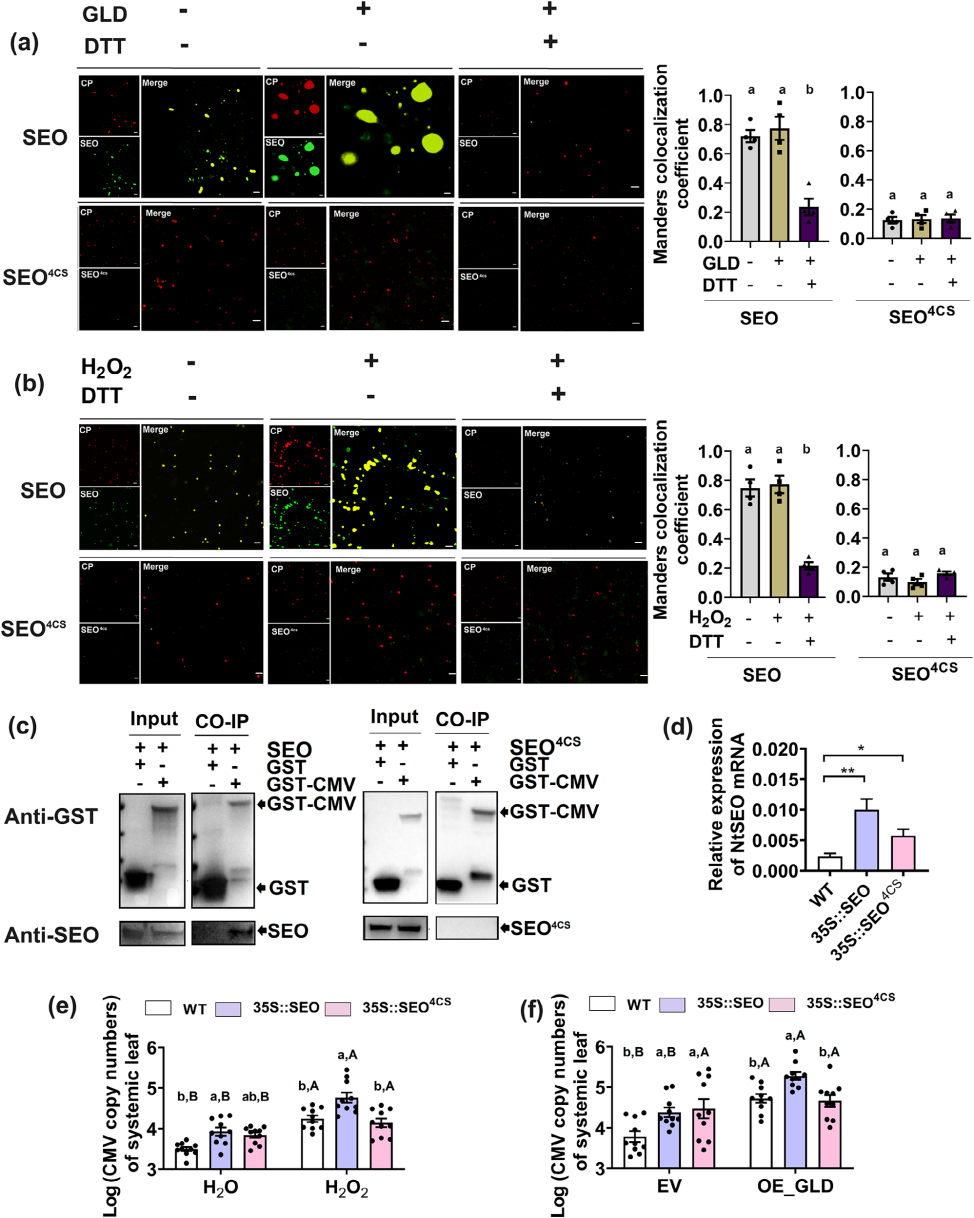
SEO aggregation triggered by an oxidative environment facilitated co‐aggregation with CMV CP and CMV systemic infection. a,b) Representative confocal microscopy images showing the effects of redox reagents, including GLD, H_2_O_2_, and DTT, on the colocalized aggregates of GFP‐SEO and mCherry‐CP (*n* = 4, one‐way ANOVA). Means with different letters represented significant differences among treatments, as determined by Tukey's post‐hoc test at *p *< 0.05. Data are presented as means ± SE. Scale bar, 10 µm. c) CO‐IP assays confirmed that CMV CP could interact with SEO rather than SEO^4CS^. d) Transcript abundance of *NtSEO* in wild‐type, *35S::SEO* and *35S:: SEO* ^4CS^ plants (*n* = 4, Student's *t* test, ^*^ *p *< 0.05, ^**^ *p *< 0.01). Data are presented as means ± SE. e,f) CMV copy numbers of plants in systemic leaves of H_2_O‐ versus H_2_O_2_‐infiltrated wild‐type, *35S::SEO* and *35S:: SEO* ^4CS^ plants, as well as in systemic leaves of GLD‐overexpressed versus EV‐infiltrated wild‐type, *35S::SEO* and *35S::SEO* ^4CS^ plants with artificial inoculation of CMV. Scale bar, 10 µm. Different lowercase letters indicated significant differences among three plant genotypes within the same H_2_O_2_ or GLD treatment, while different uppercase letters indicated significant differences between H_2_O_2_ and H_2_O treatments or between EV and GLD‐overexpression treatments within the same plant genotype, as determined by Tukey's post‐hoc test at *p *< 0.05 (*n* = 10, two‐way ANOVA). Data are presented as means ± SE.

The oxidative environment may facilitate co‐aggregation of SEO with CMV CP. 1 mm H_2_O_2_ and 25 ug mL^−1^ GLD did not directly affect the aggregation of CMV‐CP‐mCherry alone (Figure S9, Supporting Information), but promoted the co‐aggregation of SEO with CMV CP. In contrast, 10 mm reducing reagent DTT and mutation to the 4 cysteine residues destroyed the co‐aggregation (**Figure** **7**a,b). Moreover, CO‐IP showed that SEO could interact with CMV CP and that the cysteine residues were essential for their physical interaction (Figure 7c; Figure S10, Supporting Information).

### 2.8 SEO Was Required for the Systemic Movement of CMV and Oxidative Environment Accelerated This Process

To evaluate the function of four cysteine residues in the C‐terminus of the SEO protein in virus systemic movement, *35S::SEO* and *35S::SEO* ^4CS^ transgenic plant lines with enhanced SEO and SEO^4CS^ expression were generated (Figure 7d). H_2_O_2_ infiltration and transient overexpression of GLD increased CMV copy numbers of the systemic leaves in wild‐type and *35S::SEO* plants (Figure 7e,f). Little difference was found in CMV copy numbers between *35S::SEO* and *35S::SEO* ^4CS^ plants without H_2_O_2_ treatment or GLD‐overexpression. *35S::SEO* plants exhibited higher CMV copy numbers in systemic leaves than *35S::SEO* ^4CS^ plants when treated with H_2_O_2_ or GLD‐overexpression (Figure 7e,f). These results suggested that virus systemic infection was promoted in *35S::SEO* plants relative to *35S::SEO* ^4CS^ plants under an oxidative environment.

Recombinant CMV tagged with a fluorescent marker GFP (CMV‐GFP) was used to determine the essential role of SEO proteins in facilitating the long‐distance movement of CMV within plants. No obvious fluorescence was observed in the systemic leaves of *irSEO* plants regardless of GLD‐overexpression and H_2_O_2_ treatments. In contrast, remarkable fluorescence was shown in the systemic leaves of wild‐type and *35S::SEO* plants. Both GLD expression and H_2_O_2_ treatments increased the number of infected leaves and the ratio of infected area to the whole plant in wild‐type plants and *35S::SEO* plants. Furthermore, more infected leaves were observed in *35S::SEO* plants than in wild‐type plants when GLD was transiently expressed (Figure S11, Supporting Information).

## 3 Discussion

**Figure 8:**
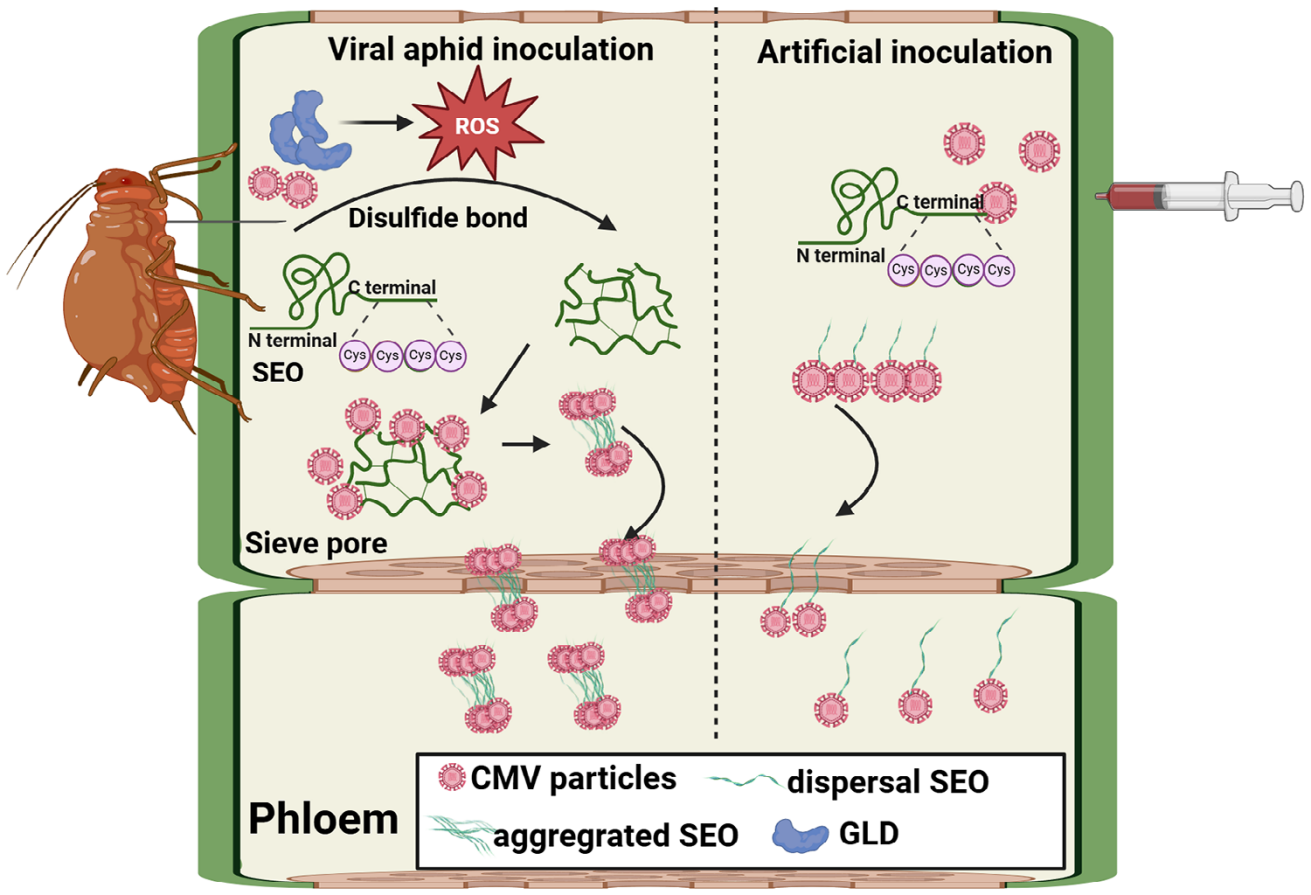
Model of the aphid salivary protein GLD in enhancing virus long‐distance movement by establishing an oxidative micro‐environment in phloem. Aphids release abundant glucose dehydrogenase (GLD) during feeding, which induces the accumulation of ROS in the phloem. This leads to the formation of intermolecular disulfide bonds among SEO proteins, promoting their transition from the dispersed state to the aggregated state. SEO can interact with CMV CP, and aphid GLD‐triggered SEO aggregation further facilitates its co‐aggregation with CMV CP. The aggregated phloem SEO proteins then load the viruses to pass through the sieve plate, thereby facilitating their long‐distance movement.

Viruses have developed and employed multiple strategies to ensure their replication and dissemination within plant and vertebrate hosts post‐infection.^[^ ^40^ ^]^ Typically, insect‐borne viruses are more infectious from the original infected site to the distal tissues when plant phloem is extensively exploited. In light of this fact, we find that the green peach aphids secrete salivary protein GLD into plant phloem during feeding, which induces the accumulation of H_2_O_2_ production. It leads to the oxidation of cysteine residues of SEO proteins in the phloem, thereby inducing a structural transformation from a randomly dispersed state to a highly ordered aggregated state. Our results further demonstrate that aggregated SEOs facilitate co‐aggregation with CMV CP under an oxidative environment, enabling the virus to cross the sieve plates. Taken together, this study highlights the coordination between a plant virus and its insect vector in facilitating long‐distance viral movement (**Figure** **8**).

Phloem maintains precise, fast, and highly dynamic adjustment mechanisms for ROS homeostasis. It can be disturbed by salivary proteins released from phloem feeders.^[^ ^41^ ^]^ Aphid‐induced ROS accumulation has been widely observed in both compatible and incompatible interactions with plants. Previous studies have shown that rapid and transient accumulation of ROS is typically associated with incompatible interactions between plants and aphids, whereas a delayed and mild ROS accumulation is most often observed in compatible interactions. For instance, a non‐tobacco adapted lineage of *M. persicae* induces stronger H_2_O_2_ accumulation in phloem than a tobacco‐adapted lineage.^[^ ^29^ ^]^ Bursts of highly reactive H_2_O_2_ in phloem lead to cellular damage as well as the formation of lignin and phenol, thereby suppressing aphid phloem feeding.^[^ ^42^, ^43^ ^]^ In contrast, plant oxidative reactions to compatible aphid infestation result from oxidative enzymes in saliva that usually facilitate the infestation process. GLD is one of the most highly abundant oxidoreductases in aphid saliva, and typically catalyzes the oxidation of the first hydroxyl group of glucose using flavin adenine dinucleotide rather than O_2_ as the primary electron acceptor.^[^ ^44^ ^]^ This explains why GLD exhibits lower oxidase activity and causes only a mild activation of ROS in phloem. Consistently, this study shows that few ROS‐related genes are significantly affected in GLD‐overexpressed plants or ds*GLD* aphid‐infested plants, with the exception of *CAT* or *APX*. Nevertheless, GLD‐induced ROS contributes to basal defenses that limit the phloem feeding of aphids when associated with GLD‐expressed plants. Moreover, there are strong indications that mild ROS, rather than strong ROS, is more favorable for thiol‐based modification of cysteine residues in target proteins.^[^ ^45^ ^]^ Our data also provides evidence that aphid GLD can establish a temperate oxidative micro‐environment in phloem, which is an ideal environment for disulfide formation of SEO protein on the thiol group of cysteine residues.

Phloem SEO proteins exhibit different conformational states in evolutionarily distinct plant families.^[^ ^23^ ^]^ In legume plants, forisome‐type SEO proteins can assemble into unique giant mechanoproteins, where multiple stages and various intermolecular interactions are involved to ensure the reversible sieve element occlusion.^[^ ^46^ ^]^ Typically, forisome monomers form dimers and subsequently filaments through hydrophobic protein‐protein interactions. Evidence from MtSEO in *Medicago truncatula*, a typical forisome in legume, shows that four hydrophobic aromatic amino acids (Trp100, Tyr115, Tyr195, and Trp196) are particularly important for dimerization.^[^ ^47^ ^]^ Two filaments wrap helically around each other to form a fibril, involving noncovalent interactions.^[^ ^46^ ^]^ Several fibrils finally assemble into fibril bundles or aggregates. Intermolecular disulfide bonds formed by conserved cysteine residues in the C‐terminus stabilize the aggregated substructures of MtSEO, whereas mutation of these cysteine residues leads to the disintegration of MtSEO upon Ca^2+^ application.^[^ ^25^ ^]^ Despite being less densely packed relative to forisome‐type SEO, dispersive‐type SEO in all dicots also exhibits aggregated fibrillar structures in immature sieve elements and displays dispersed states when the sieve elements mature.^[^ ^20^, ^22^ ^]^ In this study, we demonstrate that intermolecular disulfide bonds formed by C‐terminal cysteines promote the transition from dispersed fibrils to aggregated complexes of NtSEO. The initiation of NtSEO aggregation is further promoted by weak multivalent interactions among intermolecular IDRs in the N‐terminus, while an enhancement of co‐aggregation with NtSEO‐interactive proteins CMV CP is also observed. These results reveal that the co‐aggregation of NtSEO with CMV CP under an oxidative environment enables viral particles to form mobile aggregates that can pass through the sieve plates.

Theoretically, phloem occlusion confers efficient and robust resistance against phloem‐sucking insects due to plugging with SEO‐assembled fibril‐like structural proteins at sieve plates, which block the translocation of photoassimilates.^[^ ^48^ ^]^ To consistently absorb from the phloem of legumes, saliva effectors released by *Megoura viciae* counteract the transformation of forisome, thereby preventing phloem occlusion.^[^ ^19^ ^]^ Interestingly, our results find that aphids are difficult to ingest phloem from *irSEO* plants relative to wild‐type plants, suggesting SEO is required for aphid phloem feeding, rather than merely being involved in the physical barrier of the phloem. Similarly, another study shows that *M. persicae* fed on *AtSEOR1* and *AtSEOR2* mutants with attenuated SEO filament formation show a decreased fecundity fitness in terms of reduced reproduction and shortened reproduction period, compared to those fed on wild‐type plants.^[^ ^49^ ^]^ The negative effect of *irSEO* plants possibly results from the absence of SEO filaments and high turgor of sieve tubes, which is unsuitable for aphid feeding.

Salivary effectors of insect vectors can enhance the post‐transmission infections of insect‐borne viruses, including cell‐to‐cell movement and long‐distance phloem spread.^[^ ^10^, ^31^ ^]^ For example, salivary protein carbonic anhydrase II of *M. persicae* facilitates cell‐to‐cell translocation by accelerating intercellular vesicle trafficking,^[^ ^31^ ^]^ while GLD promotes the formation of highly ordered aggregated SEO, which in turn promotes the systemic movement of CMV by interacting with CMV CP. It is likely that the aggregation of SEO proteins triggered by aphid GLD exert mechanical pressure on the wall of the sieve pore, thereby enlarging the pore diameter and promoting the virus translocation efficiency in sieve elements. Furthermore, it has been shown that some viral components are involved in viral spread within plants. Typically, viral movement proteins control cell‐to‐cell movement, while the coat protein is essential for long‐distance transport.^[^ ^8^, ^50^, ^51^ ^]^ These two spread routes for insect‐borne viruses are significantly modified by different salivary effectors of insect vectors, which shape the outcome of virus prevalence and incidence in plant communities.

Establishing reversible physical barriers in the vascular system of both plant and animal hosts is an efficient protective mechanism against virus systemic infection.^[^ ^52^ ^]^ Animal hosts form vascular occlusion as physical barriers to eliminate virus infection by increasing vascular coagulators, including platelets, D‐dimer levels, factor VIII, and fibrinogen.^[^ ^53^, ^54^ ^]^ These coagulators, however, act as a double‐edged sword during viral infection.^[^ ^55^ ^]^ Platelets can shelter virus particles from the host immune system or even serve as a virus carrier in the circulation system.^[^ ^56^ ^]^ Here, we have shown for the first time that the plant phloem occlusion factor SEO can interact and facilitate viral particles to reach neighboring SEs. This study expands our understanding of how insect vectors aid in the exploitation of the plant vascular system by viruses and offers a promising target to impair the viral post‐transmission infection process.

## 4 Experimental Section

### Plant, Aphid, and Virus

*Nicotiana tabacum* (genotype: W38) plants were used for all experiments. Seedlings were grown under a 16 h (23 °C)/8 h (21 °C) light/dark photoperiod with 400 µmol m^−2^ s^−1^ active radiation intensity during the light period. A tobacco‐adapted lineage of *M. persicae* was reared on *N. tabacum* in a photoclimatic chamber for more than 30 generations before experiments were initiated. The SD strain of CMV, kindly provided by Professor Huishan Guo from the Institute of Microbiology, Chinese Academy of Sciences, was originally isolated from tobacco grown in the Shandong Province of China.

### Saliva Collection and Concentration

Number of 50 adult *M. persicae* were transferred from plants to a PVC tube covered with a sachet made of two layers of parafilm (Brand, Wertheim, Germany) containing 150 µL of sterilized water between the layers. The aphids were kept under light at 23 °C for 24 h. 600 µL solution per sample collected from ≈200 *M. persicae* was subjected to vacuum freeze–drying. Then, 20 µL of water was added to each sample, and the protein concentration was about 10 µg µL^−1^ for the following leaf infiltration assays.

### Virus Infection Efficiency and Titer Determination

The CMV copy numbers were compared using an artificial inoculation method in which the purified virus was infiltrated into leaves using a needleless syringe, with and without subsequent aphid feeding. CMV was purified from infected tobacco by previous methods, and the concentrations of the viruses were determined spectrophotometrically.^[^ ^57^ ^]^ Five‐week‐old *N. tabacum* plants were used for CMV inoculation. 50 µL of purified CMV virions at a concentration of 200 ng µL^−1^ were artificially infiltrated into the third mature leaf (the local leaf) from the base of the shoot by syringe without a needle. The fourth mature leaf from the base of the shoot was selected as the systemic leaf. Subsequently, 20 adult aphids were caged on the inoculated leaf for 2 h before their removal, and virus‐inoculated plants without aphid infestation were included as the corresponding control. The CMV copy numbers were quantified 7 days post aphid‐treatment.

For co‐infiltration assays, aphid saliva was treated with proteinase K, boiled, or untreated before mixing with the virus. Specifically, 20 µL of saliva (10 µg µL^−1^) was either incubated with 2 µL of proteinase K (50 ng µL^−1^) at 37 °C for 10 min, or boiled at 95 °C for 5 min. Untreated saliva was used as a control. Each saliva sample was mixed with 50 µL of purified CMV at 200 ng µL^−1^, and the mixtures were then infiltrated into local leaves. The virus copy number in local leaves and systemic leaves was determined by RT‐PCR (see next section) 7 days post‐treatment. The experiments described were performed in at least 15 biological replicates.

### Transient Overexpression of Aphid Salivary Proteins in Tobacco Plants

To determine their role in viral systemic infection, seven salivary proteins of *M. persicae* were transiently expressed in *N. tabacum*. Their transcripts in the salivary glands of *M. persicae* were quantified by qPCR, and gene‐specific primers were listed in Table S1 (Supporting Information). More specifically, the ORFs of seven salivary proteins of *M. persicae* including *LOC111043011* (*GLD*), *LOC111035238* (*CA*), *LOC111039228* (*RR1*), *LOC111031116* (*RR2*), LOC111036182 (*CSP*), *LOC111042753* (*OBP*), and *LOC111032921* (*POD*) were individually amplified, and ligated into the binary vector pBWA(V)HS‐35S under the control of the double‐enhanced cauliflower mosaic virus 35S (CaMV 35S) promoter to generate recombinant vectors (Table S1, Supporting Information). After sequence validation, these vectors were transformed into *Agrobacterium tumefaciens* strain GV3101, which was grown in LB medium with 50 mg L^−1^ Kanamycin, 50 mg L^−1^ Rifampicin at 28 °C until the OD_600_ reached 0.6. Cells were collected by centrifugation, and cell pellets were resuspended in infiltration buffer (10 mm MES, 10 mm MgCl_2_, 200 mm acetosyringone, pH 5.7) to an OD_600_ of 1.0. Three hours later, the third leaf from the base of the shoot of tobacco at the five true leaf stage was infiltrated with *A. tumefaciens*. The infiltrated areas were collected 48 h post‐infiltration for RNA isolation and qPCR reactions. Furthermore, *β*‐actin of *N. tabacum* was used as the house‐keeping gene in qPCR, and the relative level of each target gene was standardized by *β*‐actin. Two hours post‐infiltration with *A. tumefaciens*, *N. tabacum* leaves were artificially inoculated with 50 µL of purified CMV at a concentration of 200 ng µL^−1^. EV‐infiltrated *N. tabacum* plants were used as the experimental control. A TaqMan quantitative RT‐PCR assay was used to quantify CMV copy numbers as described.^[^ ^58^ ^]^ The experiments were performed in 8 biological replicates.

### Knockdown of GLD in Aphids

dsRNAs against *GLD* (246 bp) and *GFP* (439 bp) were generated using the T7 RiboMAX Express RNAi System (Promega, Madison, WI, USA), respectively (Table S1, Supporting Information). dsRNA solutions (8 µg µL^−1^) were microinjected into the dorsal abdomen (32.2 nL per injection) using a glass needle and Nanoject II system (Drummond Scientific) at a slow injection speed. To determine *GLD* silencing efficiency, five aphids per sample were collected 48 h post dsRNA injection for further qPCR analysis. Four biological replicates were included. Meanwhile, salivary glands were dissected from 50 adult aphids per sample at 48 h post dsRNA injection for qPCR quantification of salivary gland‐specific GLD transcript. Five biological replicates were included.

### Aphid Salivary Gland Preparation and Fluorescence In Situ Hybridization

To localize the *GLD* transcript in aphid salivary glands, dissected salivary glands kept in phosphate‐buffered saline (PBS) were used for FISH assays as described previously.^[^ ^31^ ^]^ A short oligo for *GLD* (5′‐Cy3‐ CCAUUUCACUGAUAGAAUAUGACUUGACCG – 3′) was used as the probe, whereas the oligonucleotide for GFP (5′‐Cy3‐ACAAGACCCGCGCCGAGGTG – 3′) and no probe were used as controls. Glands were washed extensively with PBST (PBS/0.1% Tween 20) and mounted on mounting media (Gel/Mount; Biomeda Corporation, CA, USA) on a glass slide.

### Electrical Penetration Graph (EPG)

The feeding behavior of *M. persicae* was studied via EPG analysis (Manual‐Giga‐8d.pdf http://www.epgsystems.eu/downloads, section Manuals). All aphids were subjected to a 1 h pre‐acquisition starvation period. An 8‐channel amplifier simultaneously recorded eight individual aphids on separate plants for 8 h. The following waveform patterns were scored: non‐penetration (NP), intercellular apoplastic stylet pathway in which the insects showed a cyclic activity of mechanical stylet penetration and saliva secretion (C), short intracellular punctures (pd), secretion of saliva into phloem sieve elements at the beginning of the phloem phase (E1), and passive phloem sap uptake from the sieve element (i.e., phloem ingestion E2). The EPG analysis was based on data collected from 20 aphids for each plant or aphid treatment.

### ROS Analysis

Knockdown of GLD in aphids and overexpression of aphid GLD in plants were performed to determine the impact of aphid GLD on the expression of ROS‐related genes, as well as ROS production in both mesophyll and phloem tissues. Transcripts of *Ascorbate Peroxidase* (*APX*), *Superoxide Dismutase* (*SOD*), *Glutathione S‐Transferases* (*GST*), *Catalase* (*CAT*) in infested leaves, and *Respiratory Burst Oxidase Homolog D* (*RbohD*) in infested leaf petiole were quantified by qPCR with four biological replicates, following the protocol outlined by Guo et al. (2019).^[^ ^58^ ^]^ To localize H_2_O_2_ in mesophyll and phloem, leaves were infiltrated with fluorescent probe 2 ‘, 7 ’‐dichlorofluorescein diacetate (H_2_DCF‐DA), a fluorescent dye precursor for H_2_O_2_, in pH 7.4 phosphate buffer in a closed syringe for 10–30 s. The leaves were then placed in the dark for 45 min. Fluorescence signals in tobacco mesophyll cells and phloem were visualized using a Zeiss LSM710 laser confocal microscope (Zeiss, Germany) with the adaxial side of the leaf facing the 488 nm argon laser excitation.

### Transcriptome Sequencing and Analysis

Since aphid infestation, saliva infiltration, and GLD‐expressing leaves accelerated viral systemic movement, the involvement of shared plant pathways was identified by RNA‐seq. Three groups of experiments were designed and conducted:^[^ ^1^ ^]^ 4‐leaf stage *N. tabacum* with infestation of the third leaf from the base with twenty 4th‐instar apterous aphids for 24 h versus uninfested plants;^[^ ^2^ ^]^ infiltration of the third leaf with *M. persicae* saliva (10 µg µL^−1^ from 200 aphids) versus infiltration of water for 30 min; and ^[^ ^3^ ^]^ GLD‐expressing leaves versus EV control leaves at 48 h post‐infiltration. Three biological replicates of each group were collected (18 samples in total) for RNA extraction and subsequent RNA‐seq. The concentration and quality of the total RNA were determined using an RNA Easy Mini Kit (QIAGEN, Hilden, Germany) and by gel electrophoresis. The purified mRNA was fragmented in the fragmentation buffer, and used as templates to synthesize the cDNA. The selected size DNA fragments were amplified by PCR, and sequenced on an Illumina HiSeq 2500 sequencing machine. The clean data were obtained by removing low‐quality reads from the raw data with htseq‐count software. After quality control, paired‐end clean reads were aligned to the reference genome downloaded from NCBI (*N. tabacum*: https://ftp.ncbi.nlm.nih.gov/genomes/all/GCF/000/715/135/GCF_000715135.1_Ntab‐TN90_genomic.fna.gz). Cufflinks (v2.1.1) was used to calculate the FPKM (expected number of Fragments Per Kilobase of transcript sequence per Millions base pairs sequenced) values of mRNAs in each sample. Genes were considered differentially expressed between the two treatments if *p* value *p* < 0.05 after accounting for a 10% FDR according to the Benja‐mini‐Hochberg procedure and if a fold change in expression > 2.0 was observed.

### RT‐qPCR

The RNA Easy Mini Kit (QIAGEN, Hilden, Germany) was used to isolate total RNA from *N. tabacum* leaves, phloem tissue, and the whole body of aphids. 1 µg RNA was used to synthesize cDNA. mRNAs were quantified by RT‐qPCR, which included. *GLD*, *CA*, *RR1*, *RR2*, *CSP*, *OBP*, and *POD* from *M. persicae*, *APX*, *SOD*, *GST*, *CAT*, *RbohD*, and four SEO genes *LOC107790470*, *LOC107774440*, *LOC107809602*, and *LOC107828400* from *N. tabacum*. Gene‐specific primers were designed using PRIMER5 software (Table S1, Supporting Information). The qPCR reactions were performed with the PowerUp SYBR Green Master Mix (Applied Biosystems). RT‐qPCR reactions were carried out on the QuantStudio 12 K Flex Real‐Time PCR System (ABI, A25742) as follows: 30 s at 95 °C; followed by 40 cycles of 10 s at 95 °C, 30 s at 60 °C; and finally one cycle of 15 s at 95 °C, 60 s at 60 °C, and 15 s at 95 °C. The melting curves were used to determine the specificity of the PCR products. The house‐keeping gene *β*‐actin and *MpRpL7* were used as the internal qPCR standard to analyze plant and aphid gene expression, respectively. The fold‐changes of the target genes were calculated using the 2^−ΔΔCt^ normalization method. Four biological replicates were included, and each biological replicate contained four technical replications.

### Stable Transformation of Tobacco Plants

The sequence similarity between two SEO genes *LOC107790470* and *LOC107774440* was 97%, and the hairpin (hp) RNA required for knockdown both *LOC107790470* and *LOC107774440* gene was generated. A 245‐bp conserved fragment between *LOC107790470* and *LOC107774440* was amplified (Table S1, Supporting Information). The fragment was inserted into pCAMBIA2301 transformation vector in the antisense and sense orientations to form the hairpin RNA. To generate transgenic plants overexpressing SEO (*35S::SEO*) and mutated SEO^C701704716717S^ (*35S::SEO* ^4CS^) plants, CDS sequences of *LOC107790470* and its mutant were obtained by PCR (Table S1, Supporting Information) and ligated into pCAMBIA2301, under the control of the CaMV 35S promoter. Transgenic plants, *irSEO*, *35S::SEO*, and *35S::SEO* ^4CS^ were generated through *Agrobacterium*‐mediated transformation as described previously. Briefly, hypocotyls of seedlings were cut into 3‐mm pieces with a scalpel that had been dipped in the *Agrobacterium* suspension. After callus induction and selection, light green shoots began to develop. The callus with shoots was sub‐cultured every 3 weeks until plantlets formed, when they were transferred onto a rooting medium and sub‐cultured every 3 weeks until roots appeared. These plants were carefully removed from the media and transferred to soil for further experiments.

### Transmission Electron Microscopy

To examine whether aphid GLD affected the ultrastructure of SEO proteins in plant phloem, whole plants in the four‐leaf stage with various groups of treatments were rapidly plunged into liquid nitrogen. Four groups in total were compared, including aphid‐infested plants versus uninfested plants, plants infested with ds*GLD* aphids versus ds*GFP* aphids, GLD‐overexpressing plants versus EV plants, and DTT‐ versus H_2_O_2_‐infiltrated plants. The frozen plants were then transferred to 2.5% glutaraldehyde in acetone under −80 °C for 24 h. Then, about 1 cm vascular tissues were isolated from the petioles of infested or infiltrated leaves, fixed in 2.5% (vol/vol) glutaraldehyde in acetone for 2 h, and then incubated in 1% (vol/vol) osmium tetroxide in acetone for 2 h at 4 °C. After ethanol dehydration, the samples were equilibrated gradually in cooled acetone, embedded in Resin for 2 d, and polymerized in flat embedding molds at 60 °C for 48 h. ^[^ ^4^ ^]^ Ultrathin sections were collected on copper slot grids and stained with lead citrate for up to 15 min. Sections were examined by transmission electron microscope (FEI Tecnai Spirit120kV). Fibril density of SEO and the diameter of sieve pores were measured using Image J with four biological replicates with one image analyzed per replicate. More specifically, three regions with 0.5 µm × 0.5 µm fixed‐size area were randomly selected per image, and fibril density was quantified using the “Rectangle” tool. Similarly, three sieve pores were randomly selected for each image, and the diameters were measured by the “Straight Line” tool. The average of three measurements per image was taken as one value.

### Antibody Preparation and Western Blot Analysis

The rabbit anti‐GLD (LOC111043011), anti‐SEO (LOC107790470 and LOC107774440) polyclonal antibody was prepared by Beijing Genomics Institute (BGI), using synthetic peptide CLPFGQKKDASEKNV (GLD 28–41aa), CHIYTESRARPELQY(LOC107790470, 382–395 aa), and CSRGWNEEQEMKFK (LOC107790470, 406–418aa) as the immunogen. The rabbit polyclonal antibody against CMV coat protein was also prepared by BGI, using a purified His‐tagged fusion protein as the immunogen. The western blot was performed to detect GLD secretion in leaves with 7 days of aphid infestation, as well as SEO expression in leaves or stems without aphid infestation. A polyclonal anti‐Rubisco antibody (Abcam, Cat# ab117368) was used as a positive control. 100 µg of proteins extracted from plant leaves or stems were separated by 15% SDS‐PAGE and transferred onto a polyvinylidene fluoride (PVDF) membrane. The membrane was blocked with 5% non‐fat dry milk for 2 h at room temperature and then incubated overnight with a purified primary polyclonal antibody (1:1000 dilution). Antigen‐antibody complexes were visualized using a secondary goat anti‐rabbit IgG conjugated to horseradish peroxidase (HRP) (Invitrogen) at a 1:10000 dilution, followed by detection using Pierce ECL chemiluminescent substrate (Thermo Fisher Scientific). All experiments were repeated independently three times.

### Immunofluorescence

Localization of GLD in aphid‐infested and uninfested leaves, localization of SEO in phloem, and co‐localization of SEO and CMV CP in phloem were determined. Leaves were fixed and embedded as described before.^[^ ^59^ ^]^ One‐micrometer sections were obtained from resin blocks using a rotary microtome and placed on glass slides pretreated with Vectabond (Vector Laboratories) for immunofluorescence assays. Primary antibodies diluted 1:5 in 5% milk PBS (1×) were added and incubated at room temperature for 90 min. A polyclonal GLD antibody was used to target GLD secreted into plant leaves, while polyclonal SEO and CMV CP antibodies were used to target SEO and CMV. Calcofluor white was used to stain the cell walls. After washing, secondary antibody Alexa Fluor 555 and Alexa Fluor 647 donkey anti‐rabbit IgG (Abcam, ab150074, ab150075) were diluted 1:100 in 5% milk PBS (1×) and incubated with sections at room temperature for 60 min. Sections incubated with secondary antibody without primary antibody served as negative controls. No detectable fluorescence was observed from the samples incubated with secondary antibody alone.

### Confocal Microscopy

Images were captured with a Zeiss LSM710 laser confocal microscope (Zeiss, Germany). For DAPI and Calcofluor White imaging, the employed excitation wavelength was 405 nm, and fluorescence emission was detected with a 420–480 nm bandpass filter. Moreover, the excitation wavelength of 633 nm was used to visualize Cy5 and Alexa Fluor 647, and emission was detected with a 650–750 nm bandpass filter. The excitation wavelength of 555 and 488 nm was used to visualize Alexa Fluor 555 and GFP, and fluorescence emission was detected with 570–620 nm and 505–550 nm bandpass filters, respectively. Laser power and gain were standardized across treatments to ensure comparability. Signal specificity was confirmed using autofluorescence and no‐primary‐antibody controls imaged with identical settings. Localization of H_2_O_2_ in mesophyll and phloem (H_2_DCF‐DA staining) and localization of SEO in phloem were quantified by measuring the mean gray values within manually defined regions of interest (ROIs). FISH assays of GLD in salivary glands were quantified by measuring the mean gray values of GLD and DAPI signals within manually defined ROIs. Relative fluorescence intensity of GLD was calculated as the ratio of the mean gray value of GLD to that of DAPI within defined ROIs. Fluorescence intensity of GFP‐SEO and GFP‐SEO^4CS^ proteins in vitro was quantified by calculating integrated density values within defined ROIs.

### Expression and Purification of Recombinant GFP‐SEO, GFP‐SEO^4CS^ and mCherry‐CP

To construct the GFP‐SEO and the GFP‐SEO^4CS^ mutant protein, coding sequences of *LOC107790470* and *LOC107790470*‐SEO^4CS^ were used to generate fusion DNA fragments, which were cloned into the vector pAc5.1‐hisB (Table S1, Supporting Information). The resulting constructs were transformed into *Drosophila melanogaster* Schneider 2 cells (Invitrogen) for protein expression. The soluble fusion protein was purified by Ni‐NTA (GE healthcare) affinity beads, and subsequently purified on a Superdex 200 increase 10/300 column (SD200) cascaded into an AKTA system (GE healthcare) by following the manufacturer's instructions (Sigma‐Aldrich). To express recombinant mCherry‐CP proteins in *Escherichia coli*, the coding sequences of mCherry‐CP fusion DNA were ligated into the pGEX‐4T‐1 vector by In‐Fusion cloning (Table S1, Supporting Information). The resulting constructs were transformed into *E. coli* BL21 competent cells for IPTG inducible protein expression. The soluble fusion protein was purified with glutathione resin (Sigma‐Aldrich). The purified GFP‐SEO and GFP‐SEO^4CS^ proteins were concentrated to 10 mg mL^−1^ using an Amicon Ultra‐4 Centrifugal Filter (Millipore), and mCherry‐CP was concentrated to 1.5 mg mL^−1^ stored at ‐80 °C before the phase separation assay.

### Mass Spectrometry

For the sample pretreatment, the proteins in the buffer (50 mm Tris‐HCl, 150 mm NaCl, pH 7.4, 0.1 mm GSH) with and without 10 mm DTT for 4 h were alkylated by 25 mm N‐ethylmaleimide for 45 min at room temperature in the dark, followed by overnight pepsin digestion (pepsin:protein ratio, 1:50) at 37 °C. The digested samples were loaded into Orbitrap QExactive HF mass spectrometer coupled to an EASY‐nLC 1200 system (ThermoFisher) for liquid chromatography tandem mass spectrometry (LC‐MS/MS) analysis. The resolution of MS1 and MS2 scans were set to 120000 and 15000, respectively. Raw files were searched using MaxQuant (MQ) v1.6.17.0 against a protein FASTA file for the sequence of NtSEO (LOC107790470). The search engines pLink v.2.3 and xiSEARCH were used for disulfide linkage analysis. The n‐ethylmaleimide [C] was set to variable modification.

### Protein structure Prediction

Structural prediction of the NtSEO protein encoded by *LOC107790470* was performed using the AlphaFold 3.0 algorithm (https://www.alphafoldserver.com). Structure visualizations were created in Pymol v.2.55.5 (https://github.com/schrodinger/pymol‐open‐source), The IDR of SEO was analyzed by the “VSL2” algorithm of “Predictor of Natural Disordered Regions” (PONOR, http://www.pondr.com/).

### In Vitro Phase Separation Assay

To assess the effects of redox environment on the aggregation of GFP‐SEO and GFP‐SEO^4CS^ proteins, 10 µm GFP‐SEO or GFP‐SEO^4CS^ proteins in the buffer (50 mm Tris‐HCl, 150 mm NaCl, pH 7.4, 0.1 mm GSH, 0.5% PEG400) were incubated with different redox reagents, including untreated as native condition, 1 mm H_2_O_2_, 25 µg mL^−1^ GLD, 1 mm H_2_O_2_+10 mm DTT, and 25 µg mL^−1^ GLD+10 mm DTT. To determine the effects of redox change on the co‐aggregation of GFP‐SEO or GFP‐SEO^4CS^ with mCherry‐CP of CMV, 10 µm GFP‐SEO or GFP‐SEO^4CS^ was incubated with 10 µm mCherry‐CP under different redox conditions described above. Liquid droplets were observed using a spinning‐disk confocal microscope equipped with an iXON ultra EMCCD camera (Andor). Fluorescence of GFP and mCherry was excited at 488 and 561 nm, and detected at 500–540 and 595–630 nm, respectively.

### Fluorescence Recovery After Photobleaching (FRAP)

FRAP of GFP‐SEO condensates in vitro was performed on a Zeiss 710LSM confocal microscope using a ×60 oil objective. Individual condensate droplets were photobleached at high laser power using a 488 nm laser pulse (65%) intensity, and rapid recovery (within 250 s) of the fluorescence signals of the bleached droplet was measured. Fluorescence and immunoblotting signals were quantified by calculating integrated density values within defined regions using Image J.

Fluorescence detection of SEO in 35S::GFP‐NtSEO and 35S::GFP‐NtSEO^4CS^ plants: To generate transiently expressed 35S::GFP‐NtSEO and 35S::GFP‐NtSEO^4CS^ plant, the GFP‐NtSEO and GFP‐NtSEO^4CS^ sequence was under the control of the double‐enhanced CaMV 35S promoter and ligated into pCAMBIA2301. The recombinant vector was validated by sequencing and transformed into A. tumefaciens strain GV3101. Leaves of 35S::GFP‐NtSEO and 35S:: GFP‐NtSEO^4CS^ at the four‐to‐five true leaf stage were infiltrated with A. tumefaciens. Two hours later, the local leaves were infiltrated with EV, GLD, H_2_O, and H_2_O_2_, as well as infested with dsGFP aphids and dsGLD aphids for 2 h. Two days after Agrobacterium‐mediated transient expression, the infiltrated leaves were collected for fluorescence detection.

### SEO and SEO^4CS^ Protein Expression and CO‐IP Assays

The four Cys residues located at the C‐terminal region of SEO were replaced with Ser to generate the SEO^C701704716717S^ (SEO^4CS^) mutant protein using the Mut Express II Fast Mutagenesis Kit (Nanjing Vazyme Biotech Co. China). To express recombinant SEO proteins, the coding sequences of *SEO* and *SEO* ^4CS^ were ligated into the pQE‐80L vector by In‐Fusion cloning. The recombinant SEO fusion DNA and SEO^4CS^ DNA were expressed in a cell free system by using WEPRO7240 Core Kit (CellFree Sciences Co., Ltd). Expressed SEO and SEO^4CS^ protein were examined by western blot with anti‐SEO polyclonal antibody and stored in storage buffer (50 mm Tris‐HCl, 200 mM NaCl, pH7.4).

For GST pull‐down assay, GST and GST‐CMV CP were expressed in *E. coli* and purified using the glutathione agarose beads (GE Healthcare). GST, GST‐CMV CP, and leaf total protein extracts were incubated with 300 µL glutathione agarose beads in a buffer containing 25 mm Tris‐HCl (pH 7.5), 100 mm NaCl, and 1 mm DTT for 1 h. The beads were washed seven times with the washing buffer containing 25 mm Tris‐HCl (pH 7.5), 100 mm NaCl, 1 mm DTT, and 0.1% Trition‐X 100, followed by five washes with GST‐pull down buffer before they were resuspended with loading buffer and boiled. Samples were loaded onto 10% SDS‐PAGE gels and transferred to a nitrocellulose membrane. Membranes were blocked with 5% non‐fat dry milk and 1% bovine serum albumin (BSA) for 1 h at room temperature. Membranes were washed with PBST and incubated with SEO polyclonal antibody and rabbit antibody against GST tag diluted in PBST with 2% BSA overnight at 4 °C. Subsequently, membranes were incubated with secondary antibodies for 1 h at room temperature before scanning.

### Infectivity of CMV‐GFP in irSEO, Wild‐Type, and 35S::SEO N. Tabacum Plants

An infectious cDNA clone of CMV RNA2 (pCMV‐RNA2) was engineered to construct CMV RNA2 derivatives tagged with GFP (CMV‐GFP),^[^ ^51^ ^]^ which was kindly provided by Professor Xianbing Wang (China Agricultural University). The viral CP in CMV‐GFP infiltrated plants was confirmed by western blot. A mixture of 50 µL *Agrobacterium* cultures containing pCMV‐RNA1, pCMV‐RNA2‐GFP, and pCMV‐RNA3 (OD_600_ = 1.0) were inoculated into the local leaf of *irSEO*, wild‐type, and *35S::SEO N. tabacum* plants. Two hours later, the local leaves were infiltrated with EV, GLD, H_2_O, or H_2_O_2_. Fluorescence images of plants were taken at 4, 5, and 6 days post inoculation (dpi) under ultraviolet lamp. GFP fluorescence of local and systemic leaves, as well as the infected leaf numbers of infiltrated plants at 6 dpi were compared.

### Statistical Analyses

PASW Statistics 18.0 (SPSS Inc., Chicago, IL, USA) was used for statistical analyses. All data were checked for normality by the Wilk‐Shapiro test. Data that followed a normal distribution were presented as mean ± standard error (SE). Sample sizes (n) for each group were indicated in the figure legends and within the Results section. Student's *t* test was used for two‐group comparisons. One‐way or two‐way ANOVA was used to analyze the effect of one or two independent variables. If ANOVA was significant, all post‐hoc tests were conducted using Tukey's post‐hoc test. Asterisks denoted statistical significance between two groups (^*^ *p* < 0.05, ^**^ *p* < 0.01, and ^***^ *p* < 0.001). Parameters of aphid feeding behavior were determined by the nonparametric Mann‐Whitney U test at *p *< 0.05 due to non‐normal distribution.

## Conflict of Interest

The authors declare no conflict of interest.

## Author Contributions

H.G. designed the project, performed experiments, analyzed the data, and wrote the final manuscript; Q.S., X.L., Y.W., L.H., and D.L. performed experiments; K.Z. wrote and revised the final manuscript; Y.S. contributed to project designing and coordination, and wrote the manuscript.

## Supporting information



Supporting Information

Supplemental Movie 1

Supplemental Movie 2

## Data Availability

The data that support the findings of this study are available from the corresponding author upon reasonable request.
