# Supplementary material for: Viral Systemic Movement Is Enhanced by Alteration of a Structural Phloem Protein by the Insect Vector
Source: Adv Sci (Weinh). 2025 Sep 12;12(45):e06990. doi: 10.1002/advs.202506990 (PMC12677703; doi:10.1002/advs.202506990)
Supplement: Supplementary file 1 — Supporting Information [file ADVS-12-e06990-s001.pdf]

Supporting Information

Viral systemic movement is enhanced by alteration of a structural phloem protein by the insect vector

Table S1. Primer sequences used for quantitative PCR and DNA constructs.

Gene	Primer sequence（5'-3'）	Modification	Purpose
35S::POD	*F: CGAT*GGTCTCACAACATGACGCTGAACAAAATGGTCG<br>*R:* CAGTGGTCTCATACATTATTTCTTCTTGTAATCTTCTTG	*Bsa I*<br>*Bsa I*	*DNA construct*
35S::GLD	*F:CGATGGTCTCACAACATGGACACATTCAGGACGTTGTGG*<br>*R:* CAGTGGTCTCATACATTTAGAATTATCATCCGATGATT	*Bsa I*<br>*Bsa I*	*DNA construct*
35S::CA-II	*F:CGAT*GGTCTC*ACAACATGTTTAAAGTATTCTTATTCTTATT*<br>*R: CAGT*GGTCTC*ATACAGACATAACAAAGTGGCCGATTATT*	*Bsa I*<br>*Bsa I*	*DNA construct*
35S::RR1	*F: CGAT*CGTCTC*ACAACATGAACACTTTGGTAGTGTTAGTA*<br>*R: CAGT*CGTCTC*ATACATTACTTCTTTTTGGAGTCTTCTGG*	*Bsa I*<br>*Bsa I*	*DNA construct*
35S::RR2	*F: CAGT*GGTCTC*ACAACATGAAGGTATTCATCATTCT*<br>*R: CAGT*GGTCTC*ATACATCACTTATCGTCGTCATCCT*	*Bsa I*<br>*Bsa I*	*DNA construct*
35S::CSP	*F: CAGTGGTCTCACAACATGAACACACTTCTCCTAGCAGTTG*<br>*R: CGATGGTCTCATACAGGGGTCATATTTTTGTTCAAGTTGT*	*Bsa I*<br>*Bsa I*	*DNA construct*

35S::OBP	*F: CAGTCACCTGCACAACATGAACTGCAAGGTCTTGATCG*<br>*R: CGATCACCTGCATACATTACGCGTCGAGGAATTTGTTCA*	*Bsa I*<br>*Bsa I*	*DNA construct*
GST-CMV	*F: CCGGAATTCATGGACAAATCTGAATCAACCAGTGCT*<br>*R: ATAAGAATGCGGCCGCTCAGACTGGGAGCACTCCAG*	*EcoRI*<br>*NotI*	*DNA construct*
SEO^{4CS}	*F: CAGCTCGAGGCCACCATGGCAAGTCGTGCTTTG*<br>*R: CGGGCCCATATCAGTGTAGTAACGGTA*	*XhoI*<br>*ApaI*	*DNA construct*
SEO	*F：CAGCTCGAGGCCACCATGGCAAGTCGTGCTTTG*<br>*R：CGGGCCCATATCAGTGCAGCAACGGTA*	*XhoI*<br>*ApaI*	*DNA construct*
GFP-SEO	*F: CAGCTCGAGGCCACCATGGTGAGCAAGGGCGAGGA*<br>*R: CGGGCCCTCAATCAGTGCAGCAACGGTACATGAA*	*XhoI*<br>*ApaI*	*DNA construct*
GFP-SEO^{4CS}	*F: CAGCTCGAGGCCACCATGGTGAGCAAGGGCGAGGA*<br>*R: CGGGCCCTCAATCAGTGGAGGAACGG*	*XhoI*<br>*ApaI*	*DNA construct*
mCherry- CP	*F: CCGGGATCCATGGTGAGCAAGGGCGAGGAG*<br>*R: CCGGAATTCTCAGACTGGGAGCACTCTCCA*	*BamHI*<br>*EcoRI*	*DNA construct*
NtSEO-RNAi	*F:* AGACTCGAGTTGGCAAAATCTGTGGCTCT<br>*R:* AGATCTAGAAAGGCTAGTAAGTTGAGAAG	*XhoI*<br>*KpnI*	*DNA construct*

LOC107774440	*F*: ACTCCTCAGCACTGCAATCG<br>*R*: GGTGGTACAAAGACCACCTGT	*qPCR*
LOC107809602	*F*: TTCAGCATGCTTTCCTTGCC<br>*R*: TTCCATTGTTGTTGCATGGGC	*qPCR*
LOC107828400	*F*: TTTCCCCCATCCACACCAAA<br>*R*: GTCTCGGACTGCTCCAATCG	*qPCR*
NtAPX	*F:* GCCTGATGCTACCAAGGGTT<br>*R:* GCCCTTCTTTCTCCCCACTC	*qPCR*
NtSOD	*F:* TGCGTCATGCTGGTGATCTT<br>*R:* ACCAGCATTTCCAGTGGCTT	*qPCR*
NtGST	*F*: AAGCCAGAAGAGCCCTGAAC<br>*R*: GGAAGAAGCTTGTCCTCCCC	*qPCR*
NtCAT	*F*: CAACAAGGCTGGGAAAGCAC<br>*R*: TGGTGGCGTGGCTATGATTT	*qPCR*
NtRbohD	*F:* ATCGGAACCAGTGTCGGAAC<br>*R*: AAAGCCAGCTCGGGATCTTC	*qPCR*

NtPXR	*F*: CGGAGCTTTCACTCCCACTT<br>*R*: TTAGCCGCCTTCACCTTGAG	*qPCR*
MpRpL7	*F*: TTACCCAAGTTGATACAGG<br>*R*: ACAAGCAGATTTAGCACCC	*qPCR*
MpGLD	*F*: GGACGTGGACAGCGATGAAT<br>*R*: ACGCCTTTCGAAGGTTCTGT	*qPCR*
β-actin	*F*: GGGTGACGAAGCTCAATCCA<br>*R*: TGGCCTTAGGGTTGAGAGGT	*qPCR*
MpPOD	*F*: CCAGACGAGTACTGATCGCC<br>*R*: CGTGAGCGTGAGCGTTTATG	*qPCR*
MpRR1	*F*: ATCGTTACACCACCAGCCAG<br>*R*: CGGGTTTCCTCTTGGCTCTT	*qPCR*
MpRR2	*F*: ACCCAGGTCAACAAGCCTAC<br>*R*: ACAACCTTGGGTACGACTGG	*qPCR*
MpCAII	*F*: CCCGTCCCCGTATCAGTGTA<br>*R*: CGTCGCCAGATGCTTCACTA	*qPCR*

MpCSP	*F*: CGAATAGACGGCGGACAGAA<br>*R*: CGGACTGCACGGTCCATTAT	*qPCR*
MpOBP	*F*: GGACGAAGAAGCCAGTCGAA<br>*R*: CTAATAAACGCGCGTGCGAA	*qPCR*
MpGLD-RNAi	*F*: TAATACGACTCACTATAGGACCAAATCAATTCCCAGGGCT<br>*R*: TAATACGACTCACTATAGGTGGATGTAATGCTCTTCGCCT	*dsRNA*

Underlined nucleotides represent the restriction site.

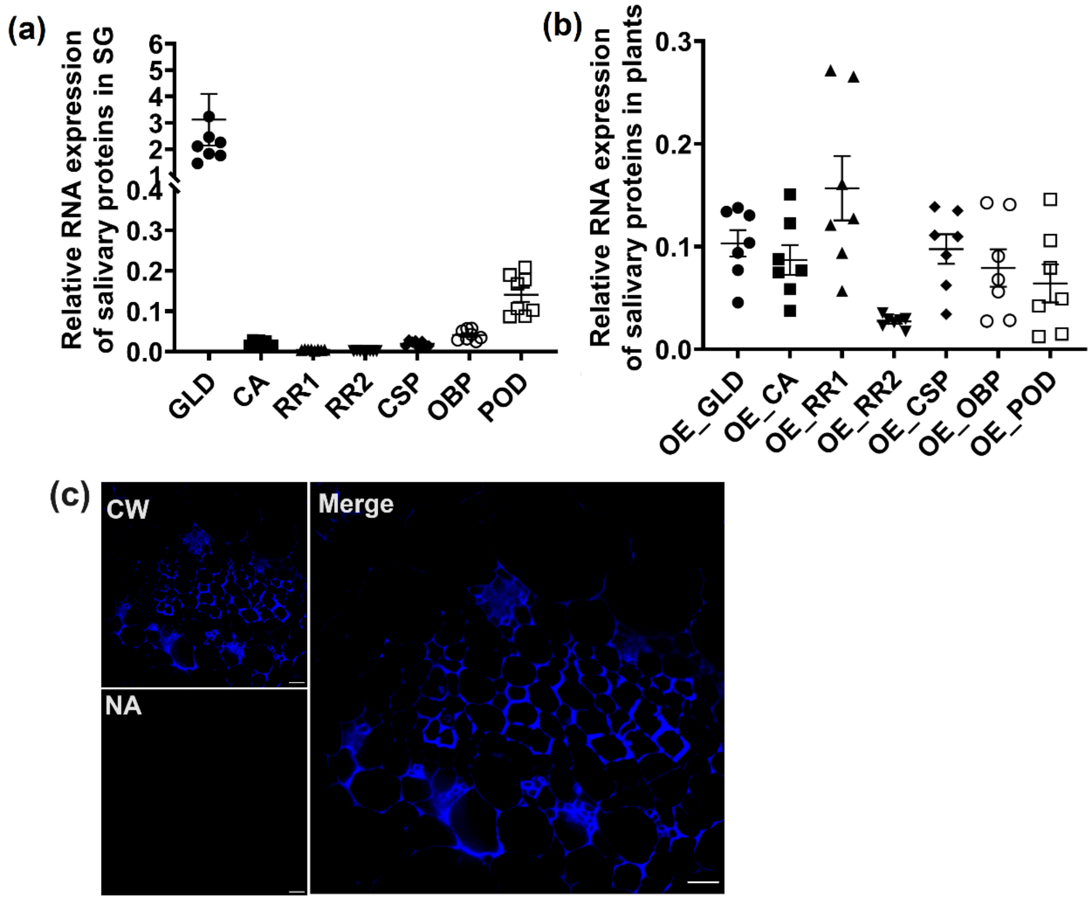

Figure S1 (a) Transcript abundance of seven salivary proteins in salivary gland of *M. persicae* (n = 8). Data are presented as means ± SE. (b) Transcript abundance of seven salivary proteins in *N. tabacum* leaves that transiently expressed the corresponding aphid gene (n=7). Data are presented as means ± SE. (c) The negative control without application of primary anti-GLD antibody for immunofluorescence assay of aphid-infested leaves. Scale bar, 20 µm. Calcufluor White was used to stain cell wall.

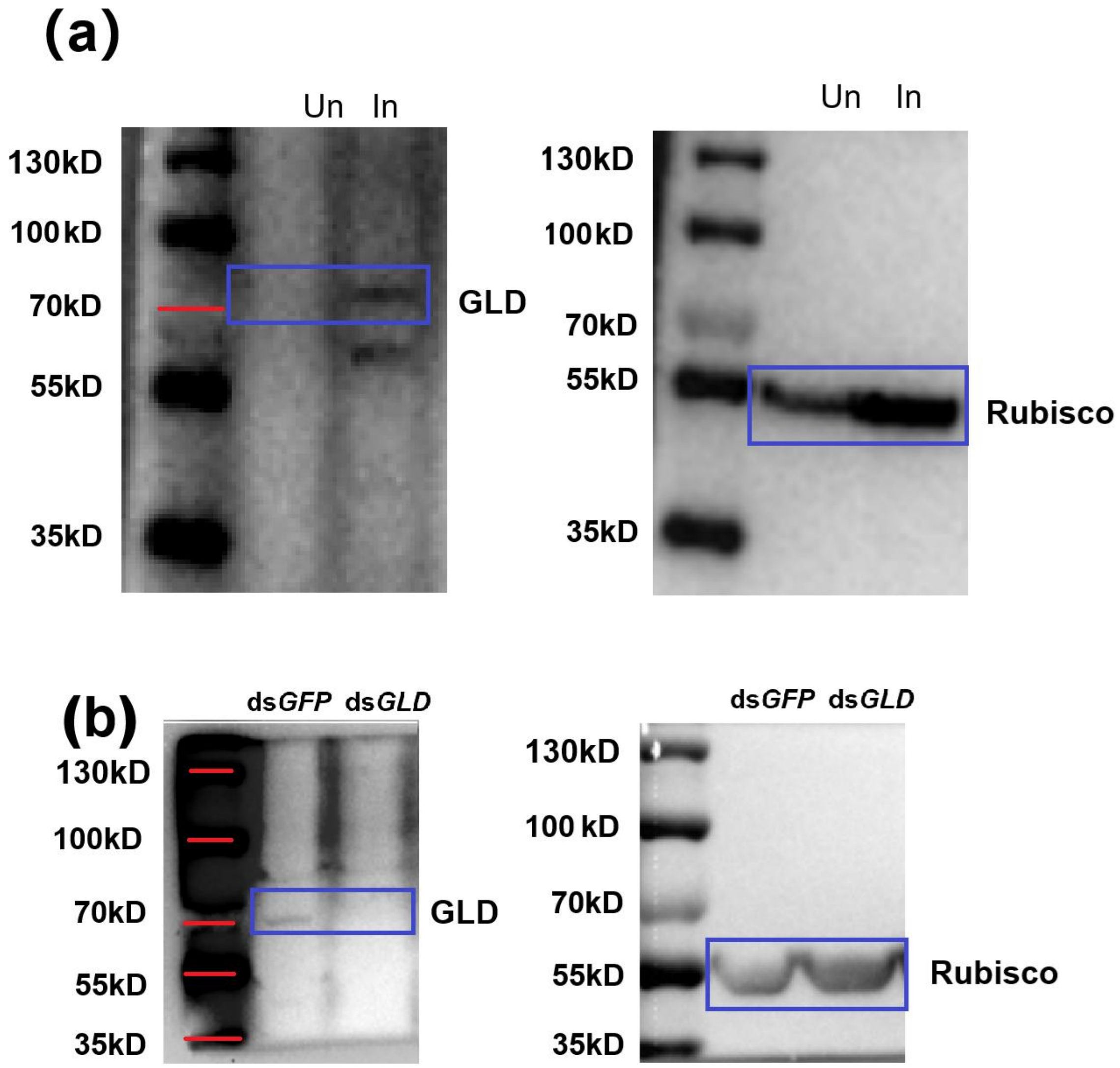

Figure S2 The full-size western blot scans of GLD in aphid-infested plants. (a) The full-size western blot of GLD in uninfested leaves and aphid-infested leaves. Lane 1: uninfested leaf; lane 2: aphid-infested leaf. The bands highlighted by blue rectangle were presented in Figure 1g. (b) The full-size western blot of GLD in ds*GLD* or ds*GFP* aphid-infested leaves. The bands within blue rectangle were presented in Figure 2d. Un: uninfested plant; In: aphid infested plant.

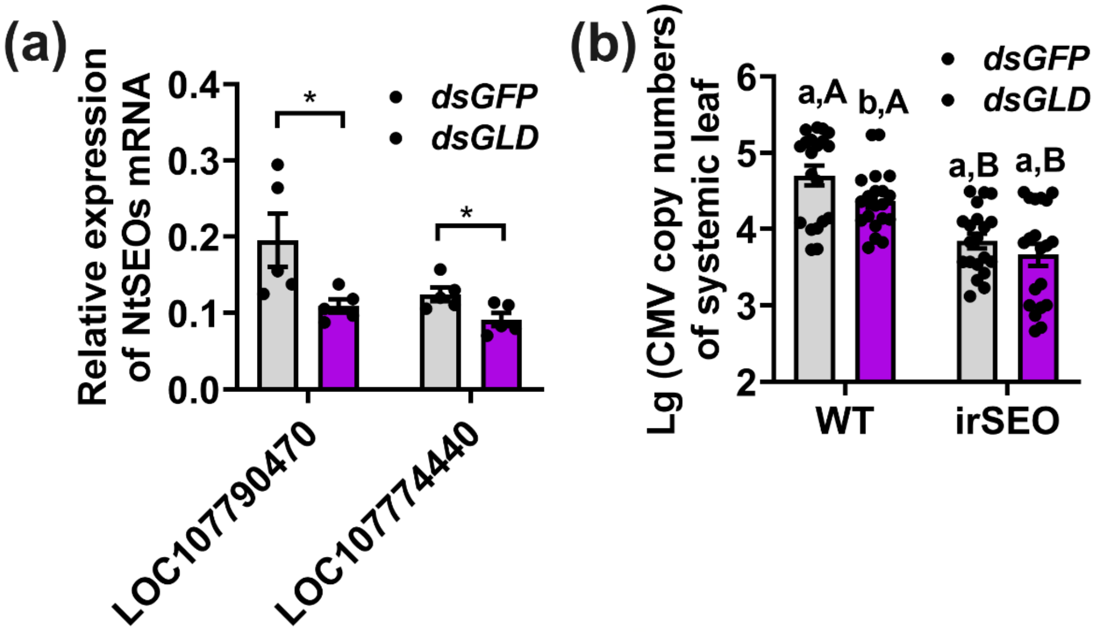

Figure S3 The transcripts of *NtSEO* and CMV copy numbers in plants when infested by ds*GFP*- and ds*GLD*-injected aphids. (a) The transcripts of *LOC107790470* and *LOC107774440* in wild-type plants when infested by ds*GFP*- and ds*GLD*-injected aphids (n=5, Student's t test, *P<0.05). Data are presented as means ± SE. (b) The CMV copy numbers in systemic leaves of CMV-inoculated wild-type and *irSEO* plants when infested by ds*GFP*- and ds*GLD*-injected aphids. Different lowercase letters indicated significant differences between dsGFP-injected aphid and dsGFP-injected aphid infestation within the same genotype plants while different uppercase letters indicated significant differences between two plant genotypes as determined by Tukey's post-hoc test at P<0.05(n=15, two-way ANOVA). Data are presented as means ± SE.

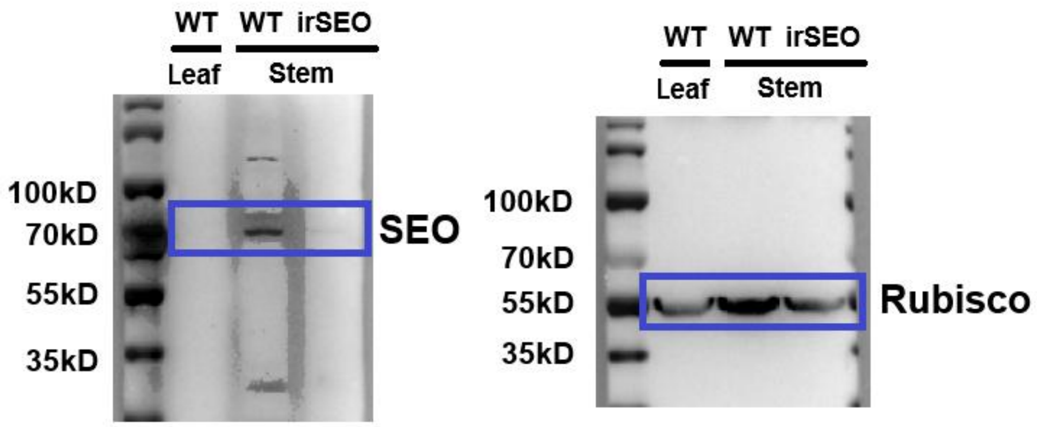

Figure S4 The full-size western blot scans of SEO in wild-type and *irSEO* plants. Lane 1: wild-type plant leaf; Lane 2: wild-type plant stem; Lane 3: *irSEO* plant stem. The bands shown in blue rectangle were presented in Figure 4d.

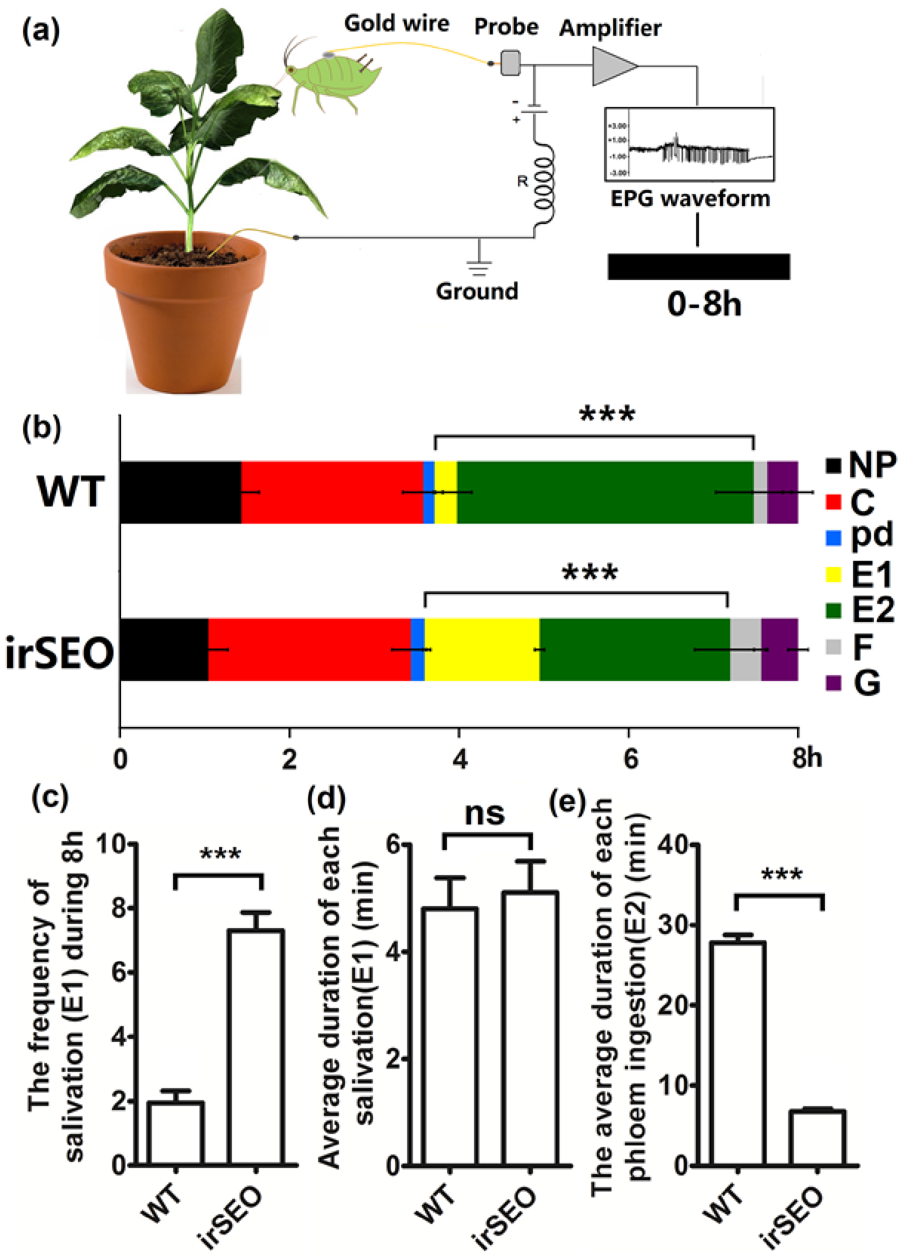

Figure S5 Aphid feeding behavior on wild-type and *irSEO* plants. (a) Schematic representation of electronic penetration graph (EPG) monitoring of aphid feeding behavior. Feeding was monitored for an 8-h periodWaveform patterns were scored: non-penetration (NP); pooled pathway phase activities in intercellular space of epidermis and mesophyll cells (C); short intracellular punctures in epidermis and mesophyll cells (pd); salivary secretion into phloem sieve elements (E1); phloem ingestion (E2); xylem injection (G); derailed stylet (F). (b) The frequency of salivation during 8h. (c) Average duration of each salivation activity. (e) The average duration of each phloem ingestion (n=20, Mann-Whitney U test, *** P<0.001. ns, no significant difference). Data are presented as means ± SE.

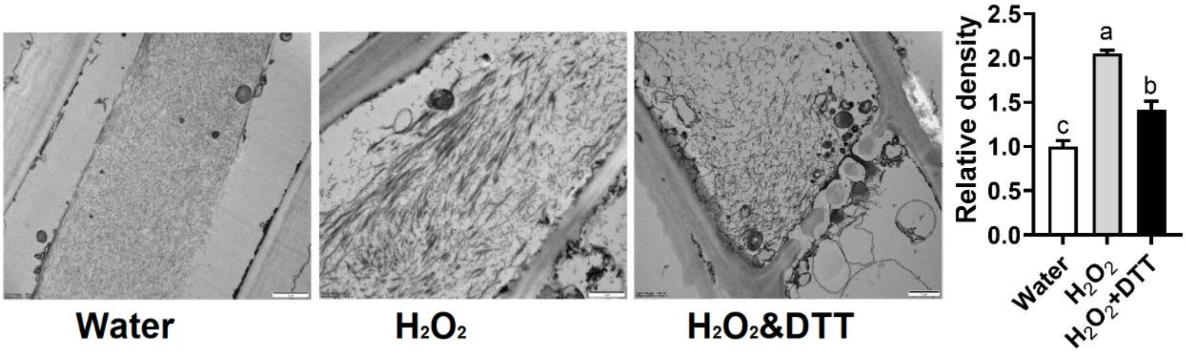

Figure S6 The ultrastructure of phloem proteins and relative density of fibril in *N. tabacum* when treated with H_2O_2 and DTT were examined using TEM. Different lowercase letters indicated significant differences among H_2O, H_2O_2 as well as H_2O_2 together with DTT treatments as determined by Tukey's post-hoc test at $P<0.05$ ($n=4$, one-way ANOVA). Data are presented as means ± SE. H_2O_2, 1 mM; H_2O_2 & DTT, 1 mM H_2O_2 + 1 mM dithiothreitol (DTT).

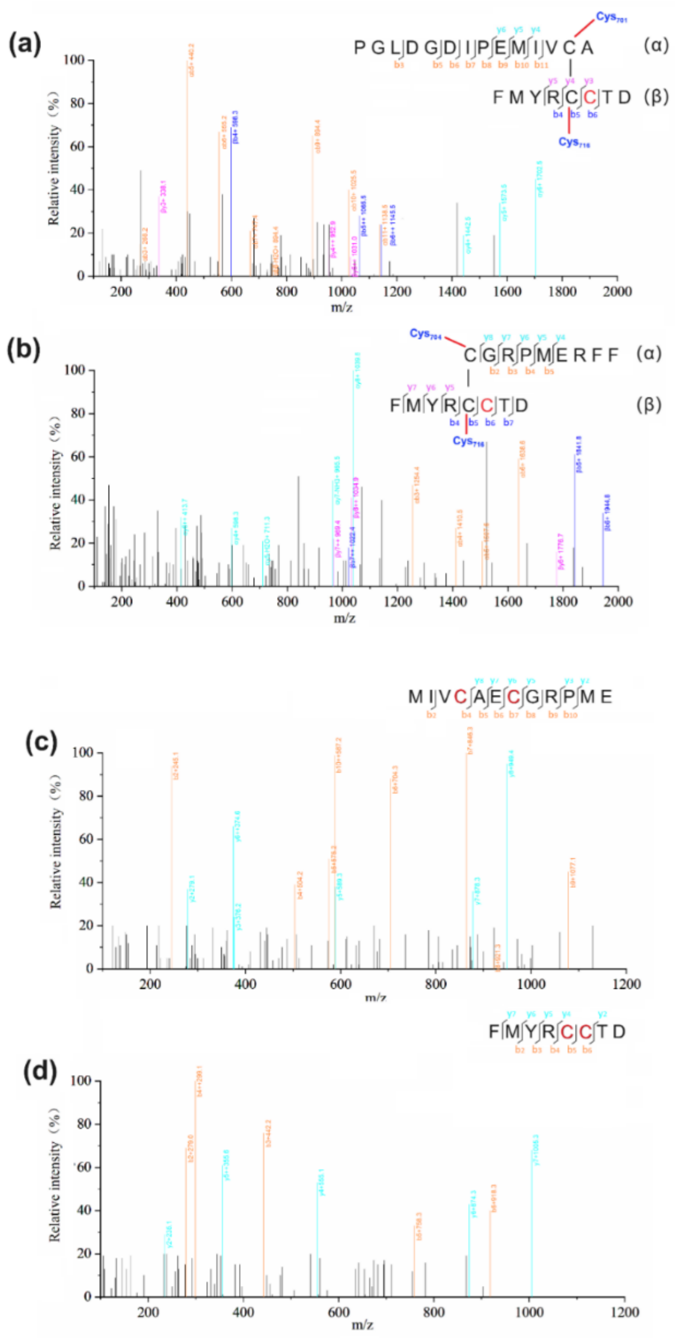

Figure S7. Identification of disulfide bonding in SEO with LC-MS/MS. Intermolecular disulfide bonds were formed via (a) C701-C716 and (b) C704-C716 crosslinks under the native condition. (c & d) No intermolecular disulfide bond was formed by the four cysteines in SEO under a reduced condition. [C] in red color represented n-ethylmaleimide modification.

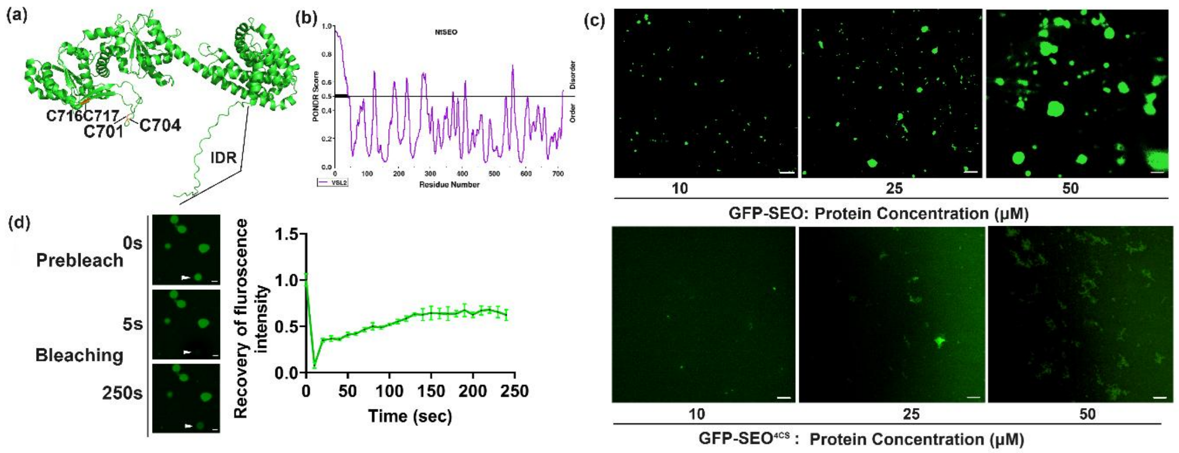

Figure S8 Characterization of SEO intrinsically disordered region (IDR), and fluorescence recovery after photobleaching (FRAP) assays. (a) NtSEO contained a 42aa intrinsically disordered region (IDR) with low-complexity domains in the N-terminus, and 4 conserved cysteine C701, C704, C716 and C717 at the C-terminus. (b) IDR was characterized by 'VSL2' algorithm of 'Predictor of Natural Disordered Regions'. (c) Purified recombinant GFP-SEO and GFP-SEO^{4CS} were seen via microscopy to form droplets of various sizes at different protein concentrations. Scale bar, 10 μm. (d) FRAP assay showing active exchange between the aggregated state and the dispersed state of SEO. White arrows indicated the bleached area in droplets. Scale bar, 5 μm. Four biological replicates were conducted in FRAP assays.

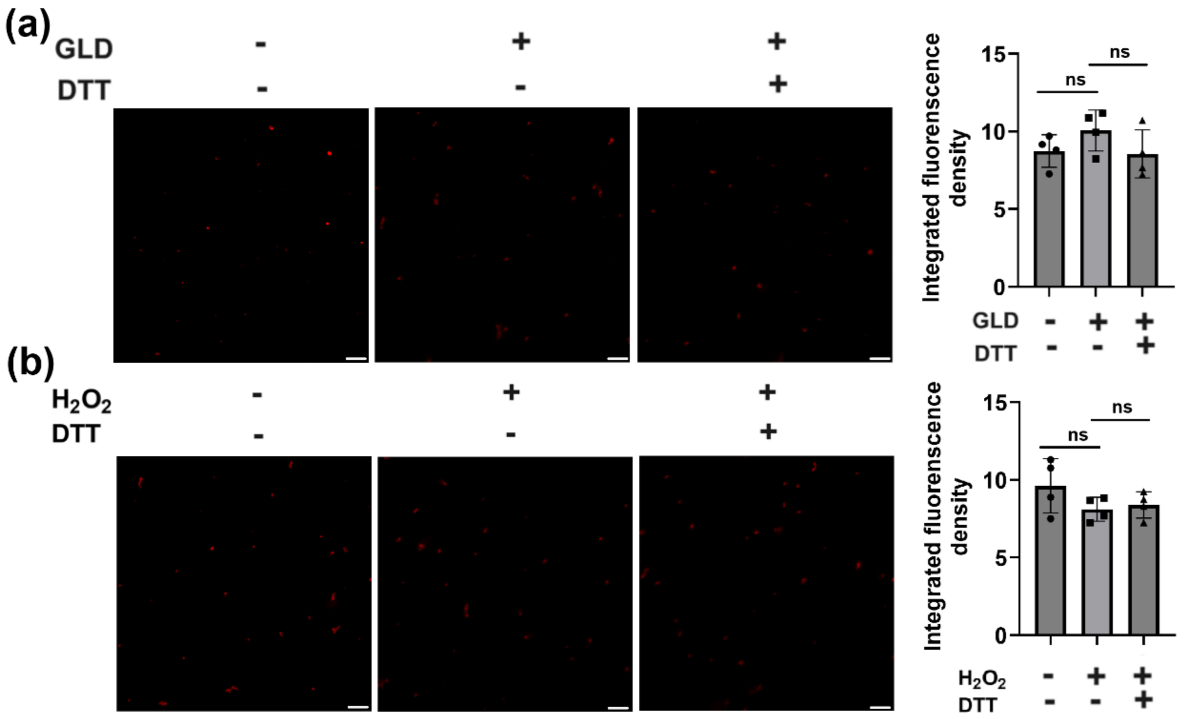

Figure S9 The effect of oxidative environment on the aggregation of coat protein (CP) of CMV. (a) Representative confocal microscopy images and quantitative data showing the effects of GLD as well as GLD plus DTT on the aggregation of mCherry-CP. Scale bar, 10 µm. (n=4, one-way ANOVA, Tukey's post-hoc test). (b) Representative confocal microscopy images and quantitative data showing the effects of H_2O_2 as well as H_2O_2 plus DTT on the aggregation of mCherry-CP (n=4, one-way ANOVA, Tukey's post-hoc test). Data are presented as means ± SE. Scale bar, 10 µm.

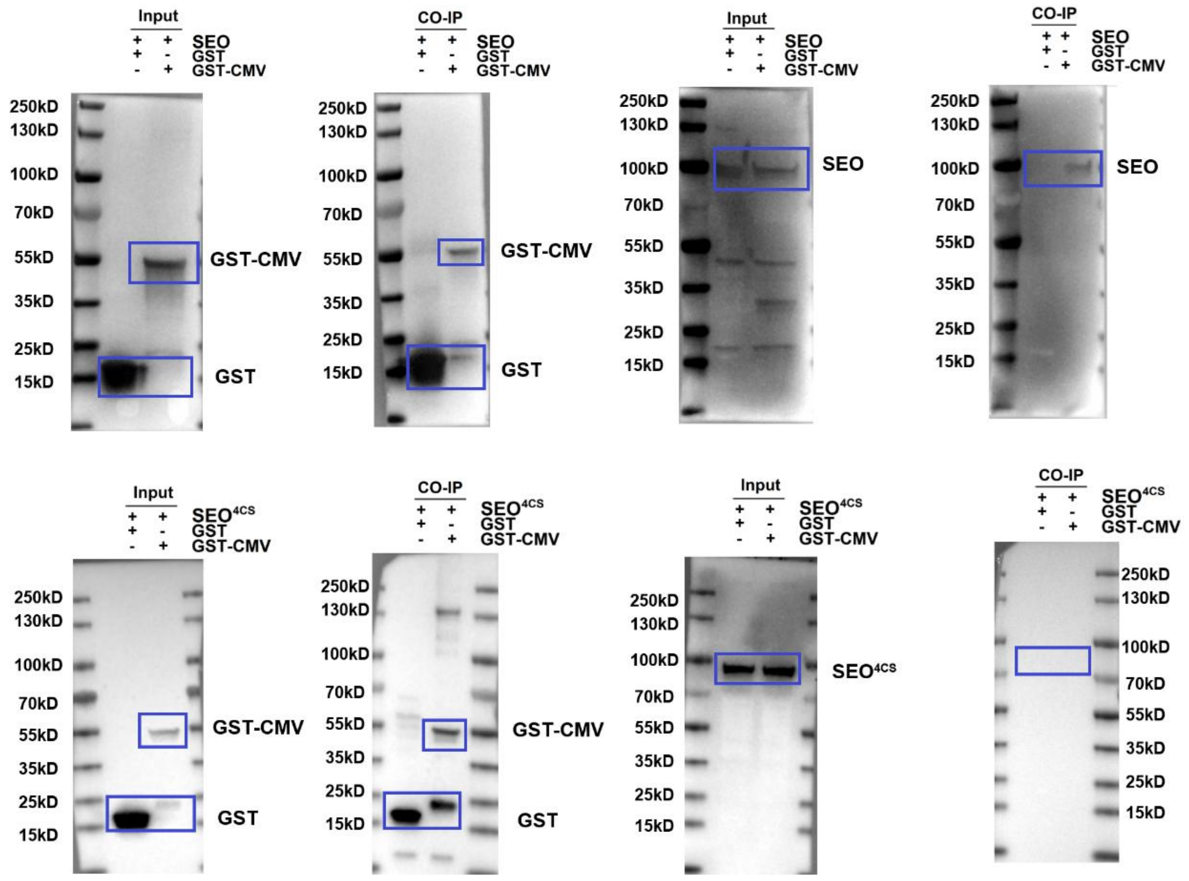

Figure S10 The full-size western blot scans of SEO and GST-CMV CP in the CO-IP assay. The bands in the blue frames are presented in Figure 7c.

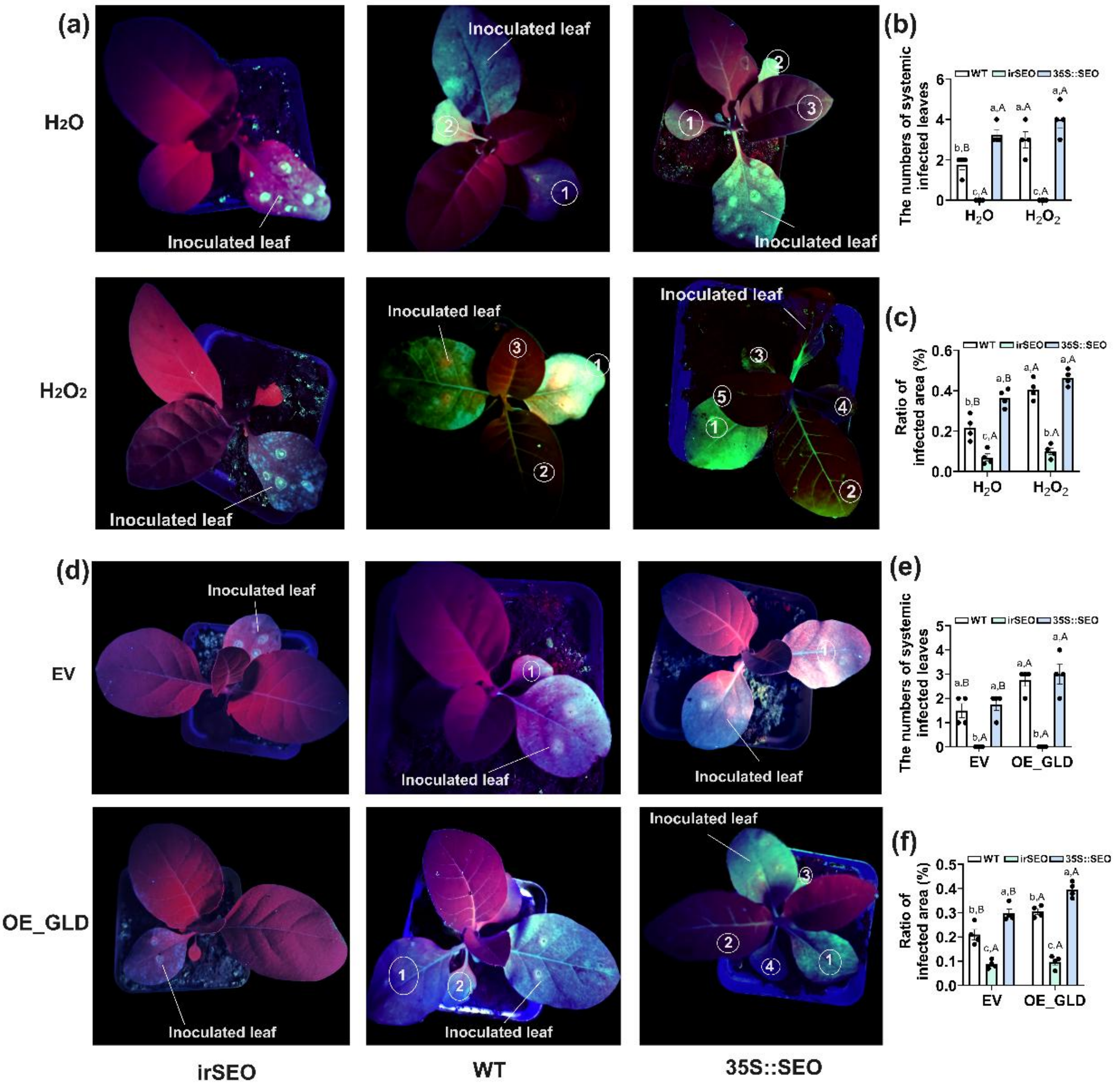

Figure S11 Infectivity of GFP-Tagged CMV (CMV-GFP) in *N. tabacum* when treated with H_2O_2 infiltration or GLD expression. (a) Fluorescence was shown in H_2O_2- or H_2O-infiltrated wild-type, *irSEO*, and *35S::SEO N. tabacum* plants at 6 days post CMV-GFP inoculation. (b & c) The number of infected leaves and the ratio of infected area to whole area were calculated in different plant genotypes with H_2O_2 vs. H_2O infiltration. Data are presented as means ± SE. Different lowercase letters indicated significant differences among three plant genotypes within the same H_2O_2 treatment and different uppercase letters indicated significant differences between H_2O_2 and H_2O treatments within the same plant genotype as determined by Tukey's post-hoc test at $P<0.05$ ($n = 4$, two-way ANOVA). (d) Fluorescence was shown in EV- or GLD-expressed wild-type, *irSEO*, and *35S::SEO N. tabacum* plants at 6 days post CMV-GFP inoculation. (e & f) The number of infected leaves and the ratio of infected area to whole area were calculated in the different plant genotypes with expression of GLD or EV. Data are presented as means ± SE. Different lowercase letters indicated significant differences among

three plant genotypes within the same GLD treatment while different uppercase letters indicated significant differences between EV- and GLD-expressed treatment within the same plant genotype as determined by Tukey's post-hoc test at $P<0.05$ ($n = 4$, two-way ANOVA).
